# Increased Tumor Growth Rate and Mesenchymal Properties of NSCLC-Patient-Derived Xenograft Models during Serial Transplantation

**DOI:** 10.3390/cancers13122980

**Published:** 2021-06-14

**Authors:** José Miguel Pardo-Sánchez, Nuria Mancheño, José Cerón, Carlos Jordá, Emilio Ansotegui, Óscar Juan, Sarai Palanca, Antonio Cremades, Carolina Gandía, Rosa Farràs

**Affiliations:** 1 Oncogenic Signalling Laboratory, Centro de Investigación Príncipe Felipe, 46012 Valencia, Spain; jmpardo@cipf.es (J.M.P.-S.); cgandia@cipf.es (C.G.); 2 Department of Pathology, University and Polytechnic La Fe Hospital, 46026 Valencia, Spain; manchenyo_nur@gva.es; 3 Department of Thoracic Surgery, University and Polytechnic La Fe Hospital, 46026 Valencia, Spain; ceron_jos@gva.es (J.C.); jorda_car@gva.es (C.J.); 4 Department of Pulmonology, University and Polytechnic La Fe Hospital, 46026 Valencia, Spain; ansotegui_emi@gva.es; 5 Department of Medical Oncology, University and Polytechnic La Fe Hospital, 46026 Valencia, Spain; juan_osc@gva.es; 6 Molecular Biology Unit, Service of Clinical Analysis, University and Polytechnic La Fe Hospital, 46026 Valencia, Spain; palanca_sar@gva.es; 7 Department of Pathology, Hospital Universitario de la Ribera, 46600 Alzira, Spain; cremades_antmir@gva.es

**Keywords:** PDX, IHC, Ki67, vimentin, ezrin, proliferation, EMT, NSCLC

## Abstract

**Simple Summary:**

Advances have been made in the study of NSCLC tumors using in vivo models, such as patient-derived xenografts (PDXs). However, the number of PDX models that can represent the heterogeneity of NSCLC between different individuals is still limited. We successfully established nine PDX mice, from which lung adenocarcinoma tumors bearing the KRAS-G12C mutation were the most frequently grafted. We show that the most aggressive tumors have a greater implantation capacity, and the success of their implantation is indicative of a poor prognosis. By using H-score to quantify cell proliferation and mesenchymal markers, we show that PDX tumors evolved towards a more proliferative and mesenchymal phenotype associated with higher protein levels of Ki67, vimentin, and ezrin, suggesting that the evaluation of their combined expression could be used as a prognostic marker to study disease progression. These PDX models provide a valuable platform for NSCLC translational research.

**Abstract:**

Non-small-cell lung cancer (NSCLC) is the leading cause of cancer death worldwide. The high mortality is very often a consequence of its late diagnosis when the cancer is already locally advanced or has disseminated. Advances in the study of NSCLC tumors have been achieved by using in vivo models, such as patient-derived xenografts. Apart from drug screening, this approach may also be useful for study of the biology of the tumors. In the present study, surgically resected primary lung cancer samples (*n* = 33) were implanted in immunodeficient mice, and nine were engrafted successfully, including seven adenocarcinomas, one squamous-cell carcinoma, and one large-cell carcinoma. ADC tumors bearing the KRAS-G12C mutation were the most frequently engrafted in our PDX collection. Protein expression of vimentin, ezrin, and Ki67 were evaluated in NSCLC primary tumors and during serial transplantation by immunohistochemistry, using H-score. Our data indicated a more suitable environment for solid adenocarcinoma, compared to other lung tumor subtypes, to grow and preserve its architecture in mice, and a correlation between higher vimentin and ezrin expression in solid adenocarcinomas. A correlation between high vimentin expression and lung adenocarcinoma tumors bearing KRAS-G12C mutation was also observed. In addition, tumor evolution towards more proliferative and mesenchymal phenotypes was already observed in early PDX tumor passages. These PDX models provide a valuable platform for biomarker discovery and drug screening against tumor growth and EMT for lung cancer translational research.

## 1. Introduction

Non-small-cell lung cancer (NSCLC) encompasses 80–85% of all lung cancer cases worldwide [1], being the most lethal (18.4%) of all cancer types, and with the highest incidence (about 2 million cases per year) [2]. According to the histology, NSCLC is classified in three subtypes—namely, adenocarcinoma (ADC or LUAD), squamous-cell carcinoma of the lung (SCC or LUSC), and large-cell carcinoma (LCC) [3,4]—with ADC and SCC being the most prevalent. Diagnosis of lung cancer at the earliest stage is strongly associated with higher survival rates, but only 16% of lung cancer cases are diagnosed at an early stage. The five-year survival rate for cases detected when the disease is still localized is 57%. However, when the disease has spread to other organs, the five-year survival rate decreases to 5% [5]. Recurrence rates vary from 35% to 55% among patients with primary stage tumors [6]. After the apparent success of initial therapy, the development of secondary tumors frequently leads to a fatal relapse. Substantial progress has been made in recent years in identifying biomarkers to select ADC patients who may benefit from targeted molecular therapy with, for instance, EGFR tyrosine kinase domain inhibitors, or the ALK receptor tyrosine kinase inhibitor [7]. Despite progress in the use of targeted therapies, the optimism arising from their application has been tempered by inconsistent responses to these therapies and the emergence of drug resistance in many patients [4]. Progress in SCC treatments has been modest, and targeted therapy in this disease has not yet been proven successful [8,9].

A better understanding of the primary tumor biology would help the design of more effective therapeutic strategies. In this sense, in vivo experimental models have been developed that allow the functional study of the patient’s ex vivo tumor [10,11,12]. These in vivo models are key tools in cancer research not only for understanding tumor biology, but also for biomarker and drug discovery validation, with mice as the main and longest used model. Patient-derived xenografts (PDXs) from NSCLC (NSCLC-PDX) consist of the implantation of a patient’s tumor cells/fragments into an immunodeficient mouse, and are currently used for lung cancer research, with advantages such as the modeling of the original cancer’s structure, and for signaling and genetic studies throughout more than 10 passages [10]. These features suggest the potential of PDXs for therapeutic screening, a handicap of traditional in vitro cancer models [13,14]. However, some drawbacks of PDXs—such as lower success of implantation (30–40%), considerable time demands (2–10 months), economic costs, limited statistical data, and high-throughput potential—must be considered altogether [15]. Beyond drug screening assays, PDXs may also be used to study oncogenic signaling pathways and cancer progression and evolution, together with the molecular mechanisms involved [16]. Among the molecular mechanisms, epithelial–mesenchymal transition (EMT) is a relevant process in pathological conditions such as cancer [17], especially in primary tumor dissociation and tumor cell intravasation [18,19]. Currently, EMT is considered a partial process with intermediate states and phenotypic plasticity, even with different levels of EMT within the same tumor [20,21,22]. Several reports suggest that EMT is relevant in metastasis [23,24], and it requires its reversal—mesenchymal–epithelial transition (MET)—for complete colonization in distant organs [25]. EMT is also associated with other characteristics of advanced tumors, such as pluripotency [26,27], the presence of cancer stem cells (CSCs) [28], and chemoresistance [21,22,29,30]. The transcriptional program of EMT is better described than its morphological manifestation [31]. At the morphological level, four main changes occur during EMT: (1) a decrease in intercellular junctions; (2) a loss of apical-basal polarity, and the appearance of front-rear polarity; (3) a reduction in the expression of epithelial markers, and an increase in the expression of mesenchymal markers; and (4) a reorganization of the actin cytoskeleton, with prominence of stress fibers, for motility purposes [18,32,33,34]. A canonical mesenchymal marker is vimentin, which is expressed in intermediate filaments (IF) in mesenchymal cells/states, and is linked to the aforementioned cytoskeleton changes. During cancer development, the increase in vimentin protein expression is usually associated with the progression, aggressiveness, and invasive capacities of the disease [35]. Another protein involved in cytoskeleton reorganization during EMT and cancer progression is ezrin, which is involved in cytoskeleton–plasma membrane–extracellular matrix mechanical signaling [36,37]. Ezrin has been shown to regulate EMT [38], local invasion, and metastasis in lung cancer [39].

In the present study, we established PDXs from surgically resected NSCLC tumors. We performed tissue microarray and compared the protein expression of cell proliferation, EMT and lung tumor markers in the primary tumor and normal adjacent tissue with the corresponding PDX, using immunohistochemistry. Additionally, we evaluated phenotypical tumor evolution using the PDX model in successive passages.

## 2. Results

### 2.1. Generation of PDXs from Surgically Resected Lung Cancer Tumors

**Figure 1:**
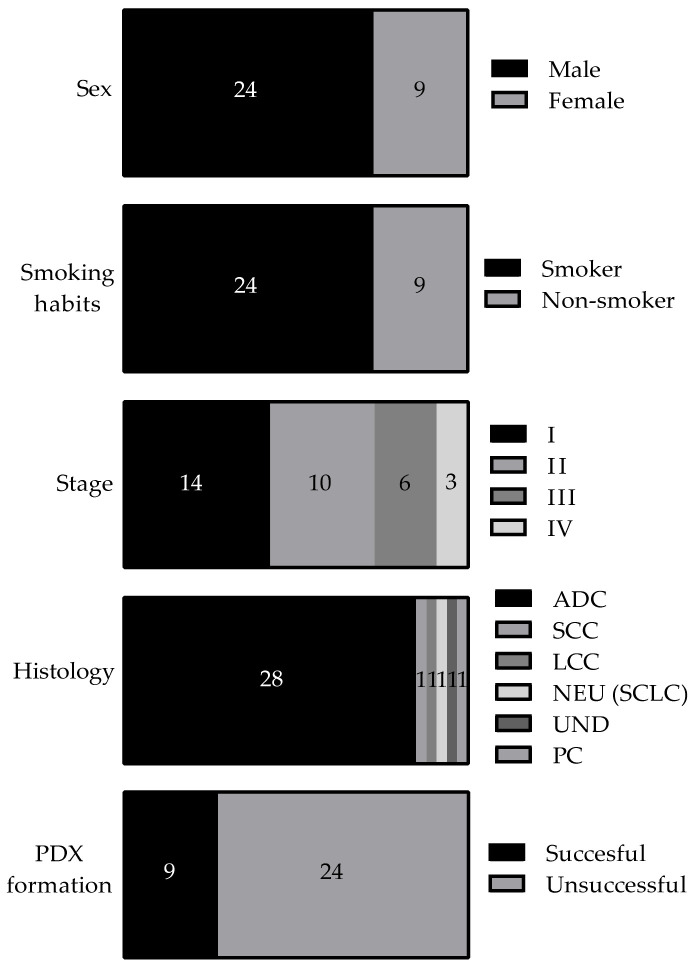
Clinicopathological characteristics of patients. ADC, smokers or ex-smokers, and males are more represented in our study. ADC: adenocarcinoma; SCC: squamous-cell carcinoma; LCC: large-cell lung carcinoma; NEU: neuroendocrine carcinoma; SCLC: small-cell lung cancer; UND: undifferentiated tumor; PC: pulmonary pleomorphic carcinoma; PDX: patient-derived xenograft.

The main clinicopathological characteristics of all lung cancer cases (*n* = 33) are presented in Table 1. The average age of participant patients was 68.3 years, and the median age was 71 years (range 42–87); 73% were males; 85% had ADC (28 out of 33 cases), with acinar, mucinous, and solid ADC being the most prevalent subtypes; and 73% were smokers (24 out of 33 cases). Most of the patients (73%) were diagnosed in early stages of the disease (14 cases in stage I, 10 cases in stage II) (Figure 1). The median follow-up was 37.4 months, the average follow-up was 32.15 months (range: 0.6–53.37), and 13 (39.4%) relapsed or died during the follow-up period. Kaplan-Meier survival analysis of different clinicopathological characteristics (Supplementary Figure S1) suggest that male patients and smokers have poorer survival rates compared to the rest of the patients. Regarding histology, patients with ADC had better outcomes.

We implanted 33 samples of lung cancer primary tumors (32 NSCLCs and 1 small-cell lung cancer (SCLC)) in mice to generate PDXs, and 9 (8 NSCLCs and 1 SCLC) out of 33 (27.3%) were engrafted successfully (Table 1, Figure 1). This rate is like other NSCLC PDX collections reported in the literature [40,41]. Supplementary Figure S2 describes the workflow we followed to generate the PDX collection from primary lung cancer tumors.

Tumors that established a PDX are also indicated. ADC: adenocarcinoma; SCC: squamous-cell carcinoma; LCC: large-cell lung carcinoma; NEU: neuroendocrine carcinoma; SCLC: small-cell lung cancer; MIA: minimally invasive adenocarcinoma of the lung; PC: pulmonary pleomorphic carcinoma; *EGFR*: epidermal growth factor receptor; *ALK*: ALK Receptor Tyrosine Kinase; PDX: patient-derived xenograft.

The focus on the generation of patient-derived xenografts (PDXs) as an in vivo model for the prognosis and evolution of patients with NSCLC implies the inclusion of PDX generation as an independent variable compared to the other clinicopathological features of the patients. In this sense, when clinicopathological variables were compared with the primary tumor engraftment success in mice, we observed that engraftment could be generated regardless of different clinicopathological variables (Table 2). However, the size of sample biopsies could be a parameter to consider for the success of the implant, since in our study samples from intermediate-sized biopsies (25–100 mm^3^) had more capacity to engraft than smaller ones (<25 mm^3^).

**Figure 2:**
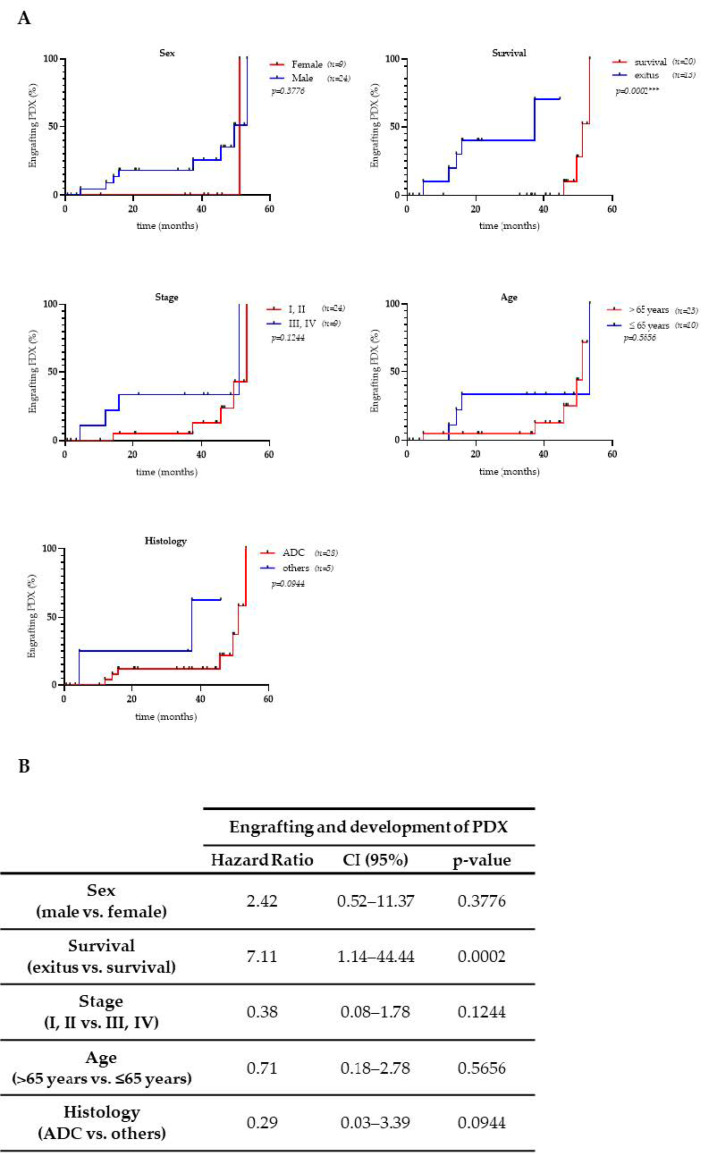
Cumulative incidence of the PDX grafting success according to clinicopathological variables. (**A**) Cumulative incidence curves showing engrafting PDX fractions throughout the follow-up period, related to each patient’s clinicopathological characteristics; (**B**) log-rank test results (Mantel-Cox) correlating PDX implantation success and the different clinicopathological characteristics. Hazard ratios, confidence intervals (95%), and *p*-values are shown. PDX: patient-derived xenograft; ADC: adenocarcinoma; CI: confidence interval.

Most samples of this study were NSCLC (32 out of 33), and most of those were ADC (28/32). From the whole collection, seven ADCs, one SCC, and one small cell lung cancer (SCLC) were successfully engrafted in mice. Cumulative incidence diagrams and hazard ratio analyses (logrank tests) showed that no significant differences were observed in the capacity of primary tumor engraftment according to histology, age, sex, or tumor stage (Figure 2). However, the survival of patients whose surgical samples had in vivo tumorigenic potential and developed PDXs was reduced (4 patients out of 9; 44.4%) compared to the samples without engraftment capacity in mice (16 patients out of 24; 66.7%), indicating a positive correlation between the progression and aggressiveness of the disease and its tumorigenic capacity and ability to engraft in mice (hazard ratio: 7.11; *p*-value: 0.0002 ***; Figure 2A, B).

Analysis of genetic alterations by massive sequencing of primary tumor biopsies that were successfully engrafted (Table 3) identified the oncogenic KRAS mutation p.G12C in ADC samples LF05, LF09, and LF15, associating these mutations with successful PDX engraftment and aggressive disease. LF15 also harbored a pathogenic mutation in ERBB2 (p.V842I), not described in lungs, but detected in gastric cancers, breast cancers, and colorectal adenocarcinomas [42]. Sample LF29 carried a germline mutation in MET (p.T1010I), which some authors associate with familial lung cancer [43], and other authors report that its pathogenicity is inconclusive [44,45,46]. LF01 carried a mutation in ERBB4 (p.L713F); however, this amino acid change is not described as oncogenic, according to COSMIC [47]. Mutations in ERBB2 (p.I767M) and MYC (p.N26S) were detected in sample LF20. The p.I767M mutation in ERBB2 has not been described as oncogenic, but has been studied in breast cancer, and appeared sensitive to conventional anti-ERBB2 drugs [48,49]. On the other hand, the p.N26S mutation in MYC is oncogenic; it has been identified in lymphomas [50] and prostate cancer [51] as well as lung cancer [52].

### 2.2. Tumor Median Latency Time Change with Increased Passage in Mice

**Figure 3:**
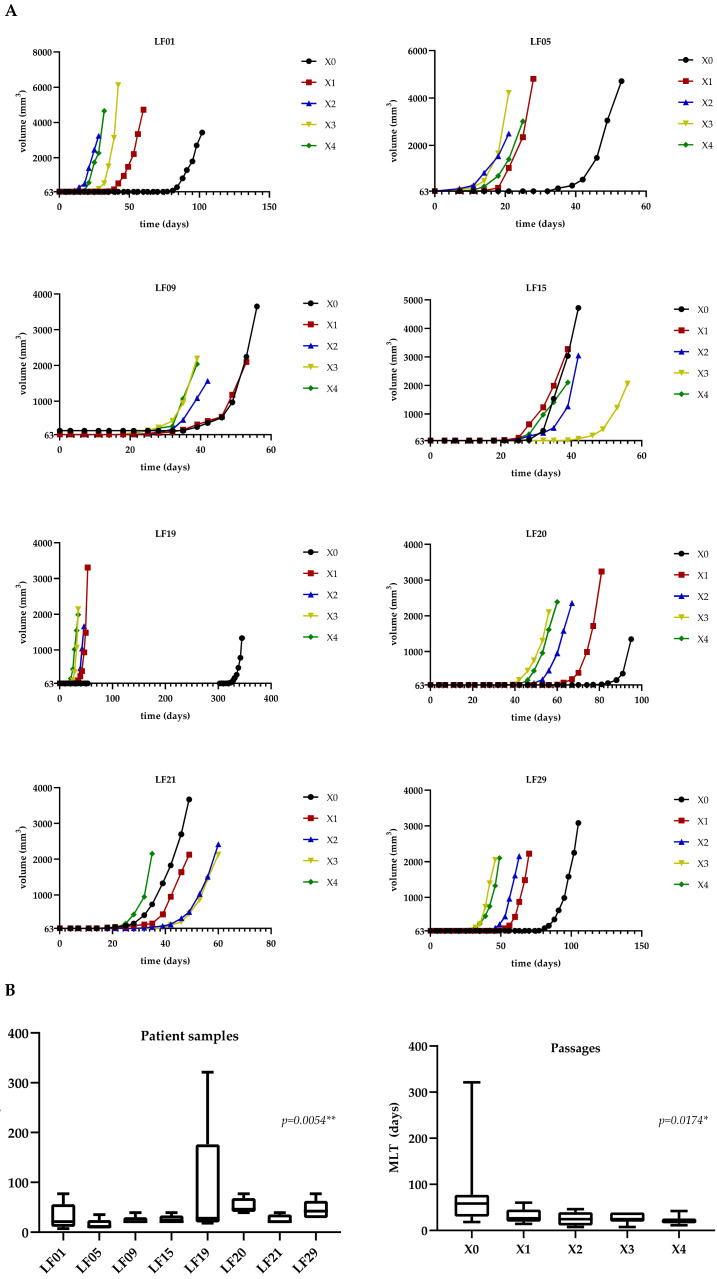
PDX tumor growth curves and median latency times. (**A**) Growth curves of PDX tumors of passages X0 to X4 from each sample are represented as volume (mm^3^) per time (days). The primary tumor implantation in mice is X0 and the last PDX tumor passage is X4; (**B**) Friedman test statistics of MLT data. (**Left panel**) Friedman test statistics of MLT data for all patient samples. (**Right panel**) Friedman test statistics of MLT data for all passages.

To study the growth characteristics of successfully implanted NSCLC tumors in mice, PDX tumors were passaged four times sequentially in mice, in addition to the original xenograft. Growth rate is often considered to be an aggressiveness, malignancy, and/or cancer status indicator when transplanting tumors into animal models, such as mice [53]. We analyzed the growth rate and latency time (the time when tumor growth is starting to be appreciated) of the eight NSCLC samples engrafted successfully in mice during four sequential passages. Figure 3A and Figure S4 show the median latency time (MLT) and growth curves from all PDX passages per sample. We observed that, on average, the growth rate of PDX tumors was similar, but median latency time (MLT) was reduced during passages. The growth of X0 tumors from seven out of eight implanted samples exhibited a MLT of 50.14 days (SD: 25.95 days). The other X0 tumor, an ADC sample (LF19), had a longer latency time of the X0 passage (321 days). In five out of seven ADC samples, including LF19 (LF01, LF05, LF19, LF20, and LF29), the passage with the longest latency time compared to successive passages was the first one (X0), suggesting that since this graft corresponds to a primary tumor section it would take more time for adaptation to the murine environment. The median latency times of the next passages were considerably shorter compared to the X0 (X1: 31.25 days ± 16.21; X2: 25.5 days ± 14.47; X3: 26.5 days ± 11.86; and X4: 21.63 days ± 9.67) and indicated the increased tumorigenic capability of NSCLC cells. Moreover, sample LF19 considerably reduced the latency time in the next passages (from 321 days in X0 to 32 in X1, 28 days in X2, 21 days in X3, and 18 days in X4). Sample LF21, which was an SCC, exhibited the shortest latency time in X0 (18 days). Low MLTs for lung SCC tumors have also been reported by other authors [54,55].

Focusing on the ADC subtypes, solid ADC (LF05, LF15, and LF29) showed a shorter MLT (28.53 days ± 18.36) through all the passages compared to acinar ADC (LF01, LF20) samples (41.8 days ± 24.26). This also correlates with the capability of solid tumors to adapt to the murine environment compared to other non-solid architectures, such as acinar ADC. However, LF19, an acinar-type tumor, behaved differently, as described above, with a very long initial latency time that grew shorter in successive passages. The mucinous lepidic ADC sample (LF09) and the SCC sample (LF21) showed MLTs for all passages of 23.4 and 25.6 days, respectively, more similar than solid ADC tumors.

The MLT data were contrasted with Friedman test statistics, showing significant differences between the MLTs of all patient samples (Friedman statistic: 20.07; *p*-value: 0.0054 **, Figure 3B) and all passages (Friedman statistic: 12; *p*-value: 0.0174*, Figure 3B). When removing LF19 because of its long MLT in X0 (321 days), differences between passages were not significant (Friedman statistic: 8.89; *p*-value: 0.0640, Supplementary Figure S3), but among patients they were still significant (Friedman statistic: 18.54; *p*-value: 0.005 **, Supplementary Figure S3).

### 2.3. Solid ADC Histological Characteristics Are Better Preserved across Patient-Derived Xenograft Passages

Previous studies showed that PDXs from NSCLC conserve morphological and molecular features from their corresponding primary tumors [54,55,56,57]. Conversely, other authors have noted that, in NSCLC PDX models, less than 60% of the PDXs preserve the traits of the original tumor [58]. To study the evolution of the PDX tumors in vivo and describe the tumor histology within the different passages, we performed tissue microarrays (TMAs) from primary lung tumor tissue, adjacent normal lung tissue, and PDX tumor tissue from passages X0 to X2. We employed hematoxylin and eosin (H&E) stain, and keratin 7 (CK7), keratin 20 (CK20), and thyroid transcription factor-1 (TTF-1) antibodies to determine the staining patterns of the lung tumors. CK7 is expressed in lung and breast epithelium, while CK20 usually marks the gastrointestinal epithelium. On the other hand, TTF-1 is expressed in type II pneumocytes and club cells, and consequently is often expressed in lung ADC (>70%) [59,60].

**Figure 4:**
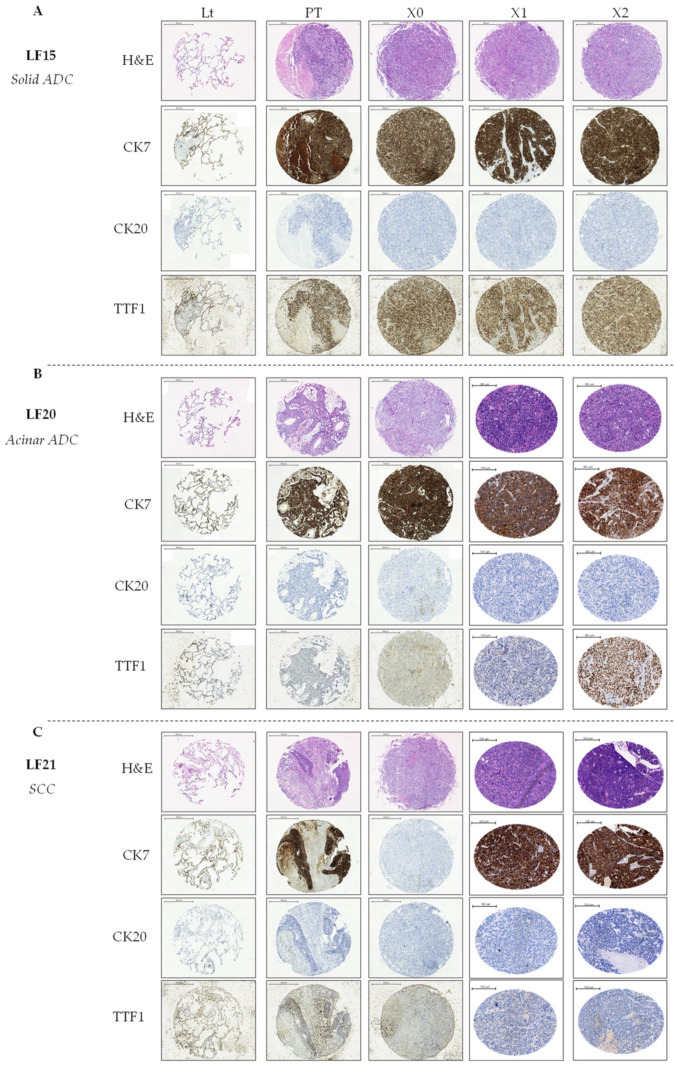
Representative images of surgically resected tumor and patient-derived xenograft (PDX) tissues on H&E and CK7, CK20, and TTF-1 staining of three different NSCLC tumors. Adjacent lung tissue (Lt), primary tumor (PT), and three PDX passages (X0–X2) are shown. All images are at 5× magnification.

The CK7+/CK20-/TTF-1+ staining pattern was observed and preserved throughout the passages (X0 to X2) in solid ADC samples LF05 (Supplementary Figure S5C) and LF15 (Figure 4A, Supplementary Figure S5G). Another solid but poorly differentiated ADC sample (LF29) preserved the original expression of the primary tumor until passage X1, and lost TTF-1 expression in passage X2 (Supplementary Figure S5O). The expression of CK7 in the solid ADC tumors increased in successive passages, as did the number of expressing cells.

The pattern of acinar and lepidic samples showed more variability through the passages. Acinar ADC LF20 maintained the CK7+/CK20−/TTF-1+ staining pattern throughout all passages (Figure 4B, Supplementary Figure S5K), while acinar ADC sample LF19 preserved the original expression of the primary tumor until passage X1 but lost CK7 and TTF-1 expression in passage X2 (Supplementary Figure S5I), and sample LF01 lost CK7 expression (Supplementary Figure S5A). Sample LF09 (mucinous, mainly lepidic ADC) expressed CK7 at the primary tumor, but lost its expression in successive passages, including the first passage (X0), although it maintained TTF-1 expression in all the passages (Supplementary Figure S5E).

Taking this into account, we observed that PDX tumors from differentiated solid ADC preserved the primary tumor histology better throughout the different passages and showed signs of dedifferentiation by increasing the expression of CK7 positive cells. Furthermore, solid ADC architecture was well preserved throughout the passages, while other types—such as acinar or lepidic—lost the expression of subtype descriptive markers in successive passages. We also observed the loss of almost all the stroma and immune cells in passage X2 (in some cases this loss was already noticeable in X1), in agreement with the observation of other authors [55,61,62], which is a limitation of this in vivo model.

An SCC sample (LF21) that also engrafted successfully in a mouse showed positive expression of CK7 and negative expression of TTF-1—which is characteristic of SCC—throughout the X0, X1, and X2 passages (Figure 4C, Supplementary Figure S5M).

All these results suggest that depending on the subtype of NSCLC there are limitations to maintaining the characteristics of the primary tumor in PDXs. In agreement with other authors [10,55], these limitations include differences in expression preservation, macrostructure, latency times, and aggressiveness of the primary tumor. Likewise, the success of the implantation of the primary tumor is due to the adaptability of the carcinoma cells, while other features that participate in the tumor microenvironment (TME) lose prominence in this in vivo model.

### 2.4. Vimentin and Ezrin Protein Expression Changes during PDX Passages

Having observed that successful sequential propagation of tumor PDXs correlated with shorter times to tumor engraftment and reduced MLTs, we analyzed the expression of protein markers associated with cell invasion and metastatic features (ezrin and vimentin), and cell proliferation (Ki67) by immunohistochemistry.

Vimentin is a canonical mesenchymal marker and may indicate differentiation and/or EMT status [63]; it is associated with cell migration and increased metastatic potential [64,65]. Ezrin is an actin-related protein that connects the cytoskeleton to the plasma membrane and is linked with local invasions [66] and metastasis [67]. Ki67 is a nuclear protein involved in cell proliferation regulation that has also been associated with tumor aggressiveness and poor prognosis [68,69,70].

We used H-score, also called histoscore [71,72,73,74]—a semiquantitative IHC analysis—to evaluate the expression of these proteins as described in the Materials and Methods section. This analysis considers the staining intensity along with the positive cells, and the value can vary from 0 to 300 [71]. A table and a bar chart including all H-scores from all PDXs at each passage for Ki67, vimentin, and ezrin expression are shown in Supplementary Table S1 and Supplementary Figures S6 and S7. The results showed that vimentin and ezrin proteins were highly expressed in the cytoplasm of most samples, while Ki67 was expressed in the nucleus (Supplementary Figure S5B,D,F,H,J,L,N,P). It has been described that while in normal tissue ezrin is correlated with a membranous expression, its cytoplasmic presence increases in cancer [75], and this is consistent with our results.

In general, the H-score showed that vimentin, ezrin and Ki67 expression remained stable in all of the samples. However, we observed that LF21 and LF29 tumors (Supplementary Figure S7) lost vimentin expression in successive passages. This coincides with the fact that the vimentin-expressing cells in their corresponding primary tumor were mostly stromal cells. While losing the original stroma of the tumor in successive passages, the vimentin staining was also lost.

**Figure 5:**
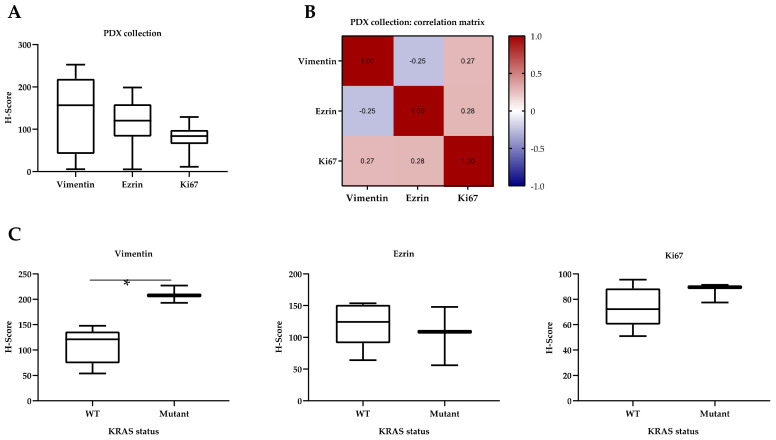
Expression of vimentin, ezrin, and Ki67 proteins in the PDX tumors. H-score analysis was used to compare the expression of proteins involved in EMT (vimentin, ezrin) and cell proliferation (Ki67) between PDX tumors. (**A**) Box and whisker plots for the H-scores of every protein in the primary tumor and the X0 to X2 passages of all PDX tumors from the 8-PDX-NSCLC collection. (**B**) Correlation matrix obtained from applying Spearman’s rank correlation coefficient to all the H-scores (*n* = 32 per protein) by pairs of proteins. (**C**) Mann–Whitney test (MW) comparison of vimentin, ezrin, and Ki67 expression with KRAS mutational status. WT: wild type; mutant: KRAS p.G12C. Coefficients are shown inside every comparison square. *: *p*-value < 0.05.

Taking into consideration all the patients and passages, H-scores of protein expression showed considerable heterogeneity between samples (Figure 5A). However, correlation matrix analysis showed a tendency of positive correlation between ezrin and Ki67 (Spearman’s coefficient (Sc): 0.279), and between vimentin and Ki67 (Sc: 0.275) (Figure 5B). Differences between the expression of proteins of interest and clinicopathological features were also studied through comparison tests. Significance was observed in the mean expression of vimentin in different samples and KRAS status, with more vimentin staining on average in samples carrying the KRAS-G12C mutation (Mann-Whitney test (MW) *p*-value: 0.0357 *) (Figure 5C). As with other authors, in this case we considered the median H-score as the cutoff value for dichotomizing the variable [73].

Then, we carried out analysis by comparing the two ADC subtypes better represented in our collection: acinar ADC (LF01, LF19, and LF20) and solid ADC (LF05, LF15, and LF29). Carcinomas are tumors of epithelial origin that often do not express vimentin because of their acute epithelial status. In agreement with this, we observed that the acinar ADC LF01 primary tumor expressed vimentin in the stroma fibers, but not in the tumor cells (Supplementary Figure S5B). However, in subsequent passages vimentin was expressed in all the tumor cells, more intensely and without specific subcellular localization. A similar pattern was observed for acinar ADC samples LF19 and LF20, which showed low expression in the X0 passage but high expression and extension of vimentin in X1 and X2 passages (Supplementary Figure S5J,L). This result is consistent with the fact that PDX tumors often dedifferentiate throughout the passages and undergo EMT at some level [76].

**Figure 6:**
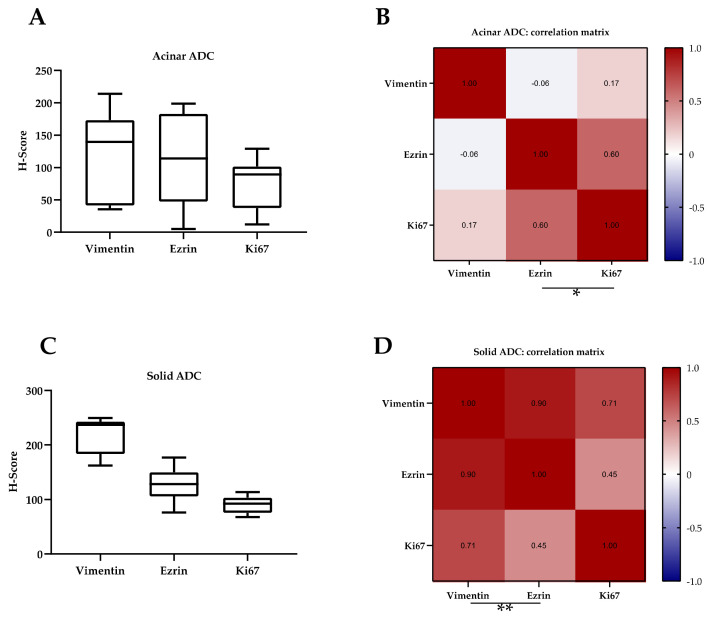
H-scores of vimentin, ezrin, and Ki67 protein expression in ADC. (**A**) Comparison of H-scores from the indicated proteins in acinar ADC patients (LF01, LF19, and LF20). The graph shows box and whisker plots for the H-scores of every protein. (**B**) Correlation matrix obtained by applying Spearman’s rank correlation coefficient to all of the H-scores (*n* = 12 per protein) of acinar ADC patients by pairs of proteins. (**C**) Comparison of H-scores from the indicated proteins in differentiated solid ADC patients (LF05, LF15). The graph shows box and whisker plots for the H-scores of every protein. (**D**) Correlation matrix obtained by applying Spearman’s rank correlation coefficient to all of the H-scores (*n* = 12 per protein) of solid ADC patients by pairs of proteins. Coefficients are shown inside every comparison square. *: *p*-value < 0.05; **: *p*-value < 0.005; ADC: adenocarcinoma.

Additionally, analysis per pair of protein expression between the acinar ADC samples (LF01, LF19, and LF20) and all the cohort data (Figure 6B) showed a significant correlation between ezrin and Ki67 (Figure 6C). Further analysis would indicate whether, in this subtype of ADC, the signaling involving these proteins is different.

On the other hand, solid ADC (LF05, LF15, and LF29) showed variable expression of vimentin. For this comparison, we only considered results from LF05 and LF15, as they were solid differentiated carcinomas, while LF29 was an undifferentiated tumor with a distinct phenotype. For instance, LF05 and LF15 (Supplementary Figure S5D,H) expressed vimentin in tumor cells other than stroma, while LF29 (Supplementary Figure S5P), which was poorly differentiated, only expressed it in the stroma of the primary tumor. LF29 PDX tumors, therefore, were like acinar LF19 and LF20 PDX tumors, compared to other solid ADC tumors. This might be due to their originally poor differentiation state.

In general, ezrin expression followed a similar pattern to vimentin throughout the passages, but with a more selective expression pattern according to the type of primary tumor. Specifically, the expression of ezrin was associated with epithelial cells (i.e., carcinoma cells), compared to the mesenchymal expression of vimentin (i.e., stroma cells/fibers). Solid ADC samples (LF05, LF15, and LF29) showed an increase in ezrin expression, in terms of intensity and number of cells throughout the passages (Supplementary Figure S5D,H,P). A similar pattern was observed for the acinar ADC tumor LF19. However, the expression of ezrin in acinar ADC samples LF01 and LF20 decreased in successive passages. In addition, subcellular location pattern changes were observed in LF19 and LF20. In these samples, the localization of ezrin in the primary tumor was in the cell membrane, and changed to the cytoplasm in passage X2, suggesting an enrichment of more aggressive cancer cells.

In contrast with acinar ADC, patients with differentiated solid ADC (LF05, LF15) showed higher expression values for vimentin, and slightly higher for ezrin (Figure 6C): vimentin (mean: 217.4; median: 237.1; SD: ±34.37); ezrin (mean: 128.3; median: 128.4; SD: ±31.01). These results suggest a possible correlation between the expression of these two proteins and the solid ADC subtype of NSCLC. This observation is reinforced by the fact that the correlation analysis showed high positive and significant correlation between vimentin and ezrin (Sc: 0.905; *p*-value: 0.005 **) (Figure 6D). Thus, PDX tumor cells may acquire more mesenchymal and invasive capacities through the successive passages. In addition, these PDX samples were derived from primary tumors that carried a driver mutation in KRAS (Table 3). Therefore, the acquisition of a more aggressive phenotype in successive passages may depend in the oncogenic KRAS signaling and may reflect the evolution of the disease in the patient.

In the unique SCC sample in the cohort (LF21), vimentin expression was lost after the X0 passage, whereas ezrin expression increased through the passages (Supplementary Figure S5N). Finally, a mucinous lepidic ADC (LF09) showed an increase in vimentin and ezrin expressions in the X2 passage (Supplementary Figure S5F).

### 2.5. Ki67 Expression in the PDX Is Associated with KRAS Mutation and Survival, and with Solid ADC Implantation Success

**Figure 7:**
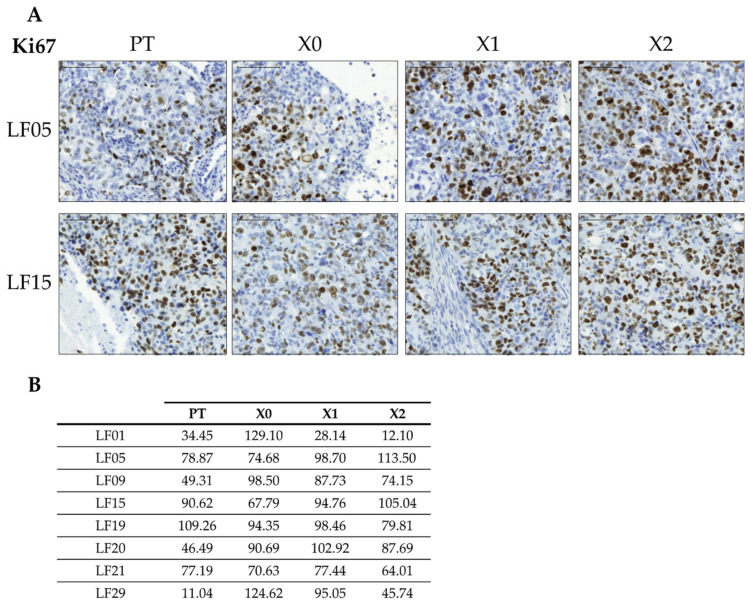
Correlation of Ki67 expression throughout the PDX passages. (**A**) Immunohistochemistry of Ki67 in samples LF05 and LF15 throughout passages X0–X2. All images at 20× magnification. (**B**) Ki67 mean H-scores of primary tumors and PDX passages of indicated samples.

Immunohistochemistry of Ki67 showed that its expression increased between the X0 and X2 passages in LF05 and LF15 PDX tumors (Figure 7A). These PDXs derived from differentiated and solid adenocarcinoma primary tumors containing a KRAS-G12C driver mutation (Table 3). NSCLC tumors with KRAS mutations show high proliferation and high Ki67 expression, with KRAS mutations in stage I lung ADC being associated with higher risk of recurrence compared to lower expressers without mutations [77]. Thus, the increased expression of Ki67 in successive passages in LF05 and LF15 PDXs suggests evolution of the tumors to higher aggressiveness (Figure 7A, B). This is also consistent with the increase in the number of cancer cells during successive passages in the PDX models, as the cells are more adapted to the murine environment during each passage [10,78]. In addition, selective pressure, as we have also observed, also benefits solid patterns [41].

Ki67 expression was higher when comparing LF05 and LF15 (mean: 90.50; SD: ±1.33) with the whole cohort (mean: 74.54; SD: ±14.78). Notably, PDX tumors derived from sample LF09—a lepidic ADC subtype and, therefore, linked to the non-aggressive lung cancer subtype—carried a KRAS oncogenic mutation and preserved the expression of Ki67 through the different passages, thus featuring enhanced aggressiveness (Supplementary Figure S5F).

**Figure 8:**
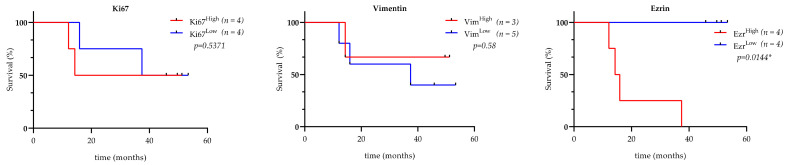
Overall survival curve (Kaplan–Meier) in relation to Ki67, vimentin, and ezrin median immunoexpression. Survival fraction (expressed in %) throughout the follow-up time is shown, related to every patient whose tumors formed PDXs (high and low expressions were obtained using the median H-scores of every corresponding protein as a cutoff).

Survival analyses using the median expression of vimentin, ezrin, and Ki67 proteins as a cutoff showed a tendency of poor prognosis for patients with tumors that expressed high ezrin and Ki67 protein levels (Figure 8). These proteins individually have been employed as markers of poor prognosis in several types of cancer. Our data suggest that it would be interesting to evaluate their combined expression levels in further studies to analyze their prognostic significance.

## 3. Discussion

The use of animal models for the study of cancer is essential in biomedical research, being especially useful in pathogenic analysis and in the design and preclinical validation of new therapies. Among these models, patient-derived xenografts (PDXs) have been especially employed as avatars of the patients with cancer, to predict therapy responses and prognosis in an almost-real-time manner [55,61]. The amplification of the patient tumor tissue in several mice for subsequent rapid analysis at different times allows a better understanding of the molecular changes that would drive metastasis and resistance to therapies. Therefore, this model can be used as an in vivo system for the testing of compounds’ anti-tumor efficacy, to guide the design of a personalized treatment, or for the identification of biomarkers. However, while it is assumed that PDXs are robust in terms of maintaining heterogeneity, genetics, and the structure of the original tumor [78,79,80,81], some authors remark that the tumor undergoes genetic and phenotypical evolution during passages in mice which can be divergent to the patient’s tumor’s evolution [82,83].

In the present study, we established 9 PDXs (7 ADCs, 1 SCC, and 1 SCLC) in NSG immunodeficient mice from 33 lung cancer patients and analyzed engraftment efficiency and tumor evolution by semiquantitative immunohistochemistry H-score analysis of tumors during 3 successive passages in mice. The percentage of grafting (27.3%) was in concordance with others reported in literature [40,41]. The clinical-pathological variables, except for the size of the tumor biopsy, did not affect the primary tumor’s engraftment success in mice. This study used a relatively small sample size, with limited statistical power; thus, future studies with larger numbers of patients would be needed to further assess the applicability of these results.

The overall survival of the nine patients providing PDX samples was lower compared to the rest of the patients (Supplementary Figure S1). Thus, a relationship between the success of heterotopic implantation in mice and the aggressiveness of the primary tumor was observed. This was reinforced by the fact that the genetic study of the primary tumors that were successfully implanted showed that they carried oncogenic mutations. Interestingly, of the seven successfully implanted ADC tumors, three contained a KRAS-G12C mutation, suggesting that aggressive tumors are those that preferentially generate grafted models. In lung ADC, KRAS mutation is found in about 32% of the cases and, of these, the KRAS-G12C mutation is the predominant, occurring in 46% of the cases. Remarkably, this KRAS-G12C mutation was the most frequently detected in our PDX collection. As great efforts are being directed towards obtaining inhibitors against this mutation [84], our PDX models may be useful to test their effect on tumor growth and predict a therapeutic response that could be translated to the clinic for patients to benefit from targeted adjuvant therapy.

We performed successive PDX passages and analyzed tumor architecture and histological patterns. In general, main histological appearances were like those of the parental tumors. However, original stromal components and immune cells were lost after several passages, suggesting that for the analysis of the interactions of the tumor microenvironment and the immune responses, the PDX models are limited. In addition, we observed that, in general, the tumor latency time in the first passage was longer than in the rest of the passages, where the tumors grew faster, indicating a tumor evolution towards greater aggressiveness during PDX passages in mice.

Apart from the patients’ clinical-pathological variables and the survival analyses to correlate them with the patients’ prognoses, the success in the engraftment of the tumors in mice can show intrinsic characteristics of the primary tumors and the tumor evolution in the PDXs. Thus, we analyzed the histological characteristics of the tumors by using a combination of markers that, in clinics, serve as diagnostic tools for the NSCLC subtype: hematoxylin and eosin stain in combination with CK7, CK20, and TTF-1. We also analyzed the expression of the canonical proliferation marker Ki67, and the EMT markers vimentin and ezrin [66]. Increasing evidence supports the role of EMT in the progression of many cancers, including NSCLC [85]. Both Ki67 [69] and vimentin [63,86] are linked to poor patient outcomes. Vimentin is an intermediate filament protein that mediates EMT and promotes cell motility [32]. Ezrin is involved in tumor invasion and metastasis [39,87]. It has been shown to play a role in TGF-β1-induced EMT in NSCLC cell lines [38] and is also associated with poor prognosis in patients with NSCLC [88,89]. We evaluated the expression of these markers in the PDX tumors during successive passages (X0–X2).

Consistent with other authors [41,54,55], our results showed that solid adenocarcinomas (ADC) are more suited to generate PDX models compared to other NSCLC subtypes (e. g.: acinar ADC), since the histochemical characteristics of the primary tumor are better preserved. In this sense, the solid ADC samples LF05 and LF15 preserved the CK7+/CK20−/TTF-1+ signatures throughout the PDX passages. The median latency times of solid ADC were shorter compared to acinar ADC PDX tumors. Acinar samples (LF01 and LF19) lost the expression of CK7 while increasing the expression of vimentin. The exchange of CK7 for vimentin could be associated with EMT by the fact that vimentin gives carcinoma cells more flexibility to change cell shape and, thus, better adapt for migration [90]. A lepidic ADC sample (LF09) lost the expression of CK7 and CK20 during the passages while preserving the expression of vimentin and Ki67. Interestingly, this tumor had a KRAS-G12C mutation, thus reinforcing the notion that aggressive primary tumors graft preferentially and suggesting tumor evolution during the passages.

Some characteristics of the primary tumor, such as differentiation and EMT status, can affect tumor evolution in successive passages in PDXs. In addition, these oncogenic processes could have different impacts on tumor evolution depending on the cancer subtype [91,92]. When evaluating vimentin and ezrin expression we observed a correlation between the expression of both proteins in our samples, except for sample LF21. In this sample, vimentin expression was observed in the stroma of the primary tumor. Since the stroma was lost in successive passages, vimentin expression was also lost. Interestingly, the expression of both vimentin and ezrin proteins was enhanced in samples derived from KRAS-G12C mutant tumors. KRAS oncogenic mutation is linked to poor prognosis due to an increase in cell proliferation, invasion, and EMT [93]. While vimentin and KRAS have been studied as interlocutors in lung ADC [94], no association have been yet established between KRAS driver mutations and ezrin. However, it has been shown that the activity of several signaling pathways—such as EGFR, Src, Akt-PI3K, and PKA—affects ezrin expression and, in addition, that ezrin regulates these signaling pathways [95]. Moreover, high ezrin expression has been associated with BRAF mutation in colorectal cancer [96]. Ezrin acts as a linker protein to transduce mechanical signals from the membrane to the cytoskeleton and promotes cell migration and invasion. Modification of ezrin proteins, such as phosphorylation, enhances Ezrin–EGFR interaction and drug resistance to erlotinib [97]. In addition, S-nitrosylation of ezrin has been shown to mediate non-small-cell lung cancer invasion and metastasis [87,89]. Thus, it is an attractive target to inhibit cancer progression and to overcome drug resistance in NSCLC.

The PDX tumors derived from KRAS-mutated samples also correlated with more proliferative phenotypes (progressive increase of Ki67 and shorter median latency times). This proliferative phenotype was also observed when ezrin was highly expressed, and progressively increased its expression and localization in the cytoplasm between the X0 and X2 passages.

## 4. Materials and Methods

### 4.1. Patient Selection and Sample Collection

Patients from La Fe University and Polytechnic Hospital (Valencia, Spain) were included in this study from October 2016 to June 2018. A total of 33 samples were collected during that time, from patients who underwent surgery according to the following criteria: resectable, non-pretreated, and lung cancer histological diagnosis. The samples were obtained from surgical specimens at the time of resection and examined by the pathologist. The necrotic tissue was removed, and the samples were transported to the laboratory in tubes filled with culture media (Dulbecco’s Modified Eagle’s Medium (DMEM-F12). Patients’ follow-ups for the purposes of this research were carried out until February 2021. Samples were collected with a sterile procedure and put in basic medium (DMEM-F12, Penicillin/Streptomycin 1%) until their arrival at the Centro de Investigación Príncipe Felipe (CIPF, Valencia, Spain), where they were processed. In every case, a lung cancer biopsy (from 25 to 100 mm^3^ size) and a normal lung-adjacent biopsy were collected for comparison purposes.

### 4.2. Biopsy Processing

Primary tumor and normal adjacent lung samples were processed in parallel. First, specimens were washed with cold PBS and minced into small portions (≈5 mm^3^). One portion was preserved in a sterile microtube and frozen at −80 °C (to preserve RNA integrity until gene expression analysis). Another portion was preserved in 4% paraformaldehyde at 4 °C, and the next day washed with PBS. This was performed for fixation and subsequent immunohistochemical analysis. The last portion was preserved on ice and immediately implanted in a mouse. The rest was minced into smaller portions (≈1 mm^3^) and enzymatically disaggregated for establishing primary cultures for further studies.

### 4.3. Mutation Analysis

The oncogenic characterization of the molecular subtypes of NSCLC was performed by massive sequencing using the Oncomine Focus Assay panel (Thermo Fisher Scientific, Waltham, Massachusetts, USA) on the Personal Genome Machine ™ platform (PGM ™; Life Technologies, Carlsbad, USA) at the Molecular Biology Unit (University and Polytechnic La Fe Hospital, Spain)—an ISO 15,189 accredited laboratory. This panel allows the detection of mutations in 35 oncogenes (*AKT1*, *ALK*, *AR*, *BRAF*, *CDK4*, *CTNNB1*, *DDR2*, *EGFR*, *ERBB2*, *ERBB3*, *ERBB4*, *ESR1*, *FGFR2*, *FGFR3*, *GNA11*, *GNAQ*, *HRAS*, *IDH1*, *IDH2*, *JAK1*, *JAK2*, *JAK3*, *KIT*, *KRAS*, *MAP2K1*, *MAP2K2*, *MET*, *MTOR*, *NRAS*, *PDGFRA*, *PIK3CA*, *RAF1*, *RET*, *ROS1*, and *SMO*), the variation in the number of copies in 19 genes (*ALK*, *AR*, *BRAF*, *CCND1*, *CDK4*, *CDK6*, *EGFR*, *ERBB2*, *FGFR1*, *FGFR2*, *FGFR3*, *FGFR4*, *KIT*, *KRAS*, *MET*, *MYC*, *MYCN*, *PDGFRA*, and *PIK3CA*), and the presence of fusion transcripts in 23 genes (*ABL1*, *AKT3*, *ALK*, *AXL*, *BRAF*, *ERG*, *ETV1*, *ETV4*, *ETV5*, *EGFR*, *ERBB2*, *FGFR1*, *FGFR2*, *FGFR3*, *MET*, *NTKR1*, *NTKR2*, *NTKR3*, *PDGFRA*, *PPARG*, *RAF1*, *RET*, and *ROS1*).

The average coverage of the panel is >1200x, the average uniformity is 95%, and the percentage of average readings in the sequences of interest (on target) is 97%. The search for variants was performed by alignment to the hg19 human reference genome using the Variant Caller algorithm. Non-pathogenic variants were filtered with the Ion Reporter software, excluding intronic variants, synonymous variants, and polymorphic variants (MAF, or minority allele frequency ≥0.01, and/or including dbSNP). Additionally, all the identified variants were reviewed in parallel using the Integrative Genomics Viewer program (IGV v.2.4; http://software.broadinstitute.org/software/igv/, accessed on 14 June 2021) and consulting different databases’ (COSMIC, TCGA, Pubmed) and programs’ predictions in silico (Polyphen, Provean, Sift, SNPS & GO).

### 4.4. Mice and Patient-Derived Xenografts

To develop xenografts in mice as in vivo lung cancer models, a small portion of the minced tumor biopsy was implanted subcutaneously. 

NOD SCID gamma (NSG) immunodeficient mice (NOD.Cg-Prkdcscid Il2rgtm1WjI/SzJ, Charles River, Wilmington, Massachusetts, USA) were transplanted subcutaneously, after a single dose of pain reliever (Buprenorphine hydrochloride, 0.6 mg/kg,), before surgery. Then, isoflurane was used as anesthetic (initial dose of 5% and a maintenance dose of 2.5%), and an incision about 2–3 mm was made in the caudal dorsal skin for implantation of a 5-mm^3^ portion of the tumor biopsy. The wound was sealed with a topical skin adhesive (Histoacryl, Braun, Melsungen, Germany). Afterwards, tumor volume (TV) was measured with a caliper every 3–4 days using the formula: TV (mm^3^) = d2 × D/2, where d and D are the shortest and the longest diameters, respectively. Animals were terminated (carbon dioxide) when tumor xenografts were 1500 mm^3^ or more. All animals that did not show tumor growth after 8 months of implantation were euthanized with carbon dioxide. To confirm mouse death, cessation of heartbeat and respiration, pale mucous membranes, and absence of reflexes were evaluated.

Portions of the PDX tumors were stored for histological (immunohistochemical) analyses and cryopreserved for further studies. Two portions (≈5 mm^3^) of the PDX tumors were used for transplantation in two new mice to establish subsequent PDX models, until the third passage was performed (X0-X2). The necrotic tissues were removed before implantation. All the PDX tumors were successfully implanted.

### 4.5. Immunohistochemistry Analyses

Fixed samples from primary tumor biopsies and PDXs (with 4% paraformaldehyde) were subjected to immunohistochemical analyses. For high-throughput screening of samples (8 patients, 5 samples each) and markers (1 stain and 7 proteins), we developed a paraffin tissue microarray (TMA) system, based on the construction of a paraffin block (recipient) for whole analyses using the MTA-1 Manual Tissue Arrayer (Beecher Instruments, Estigen, Estonia), where traditional paraffin-embedded tissue blocks acted as donors. A 1-mm needle was utilized, and the TMA protocol was performed as described in previous works [98]. Together with the hematoxylin and eosin stain (H&E, Automated H&E Staining, Dako CoverStainer, Agilent, Santa Clara, California, USA), the proteins analyzed with the TMA and the respective antibodies were as follows: keratin 7 (IR61961-2, Agilent), keratin 20 (IR77761-2, Agilent), and thyroid transcription factor-1 (TTF-1, IR05661-2, Agilent) were used as a descriptive panel for lung adenocarcinomas; Ki67 (IR62661-2, Agilent) was used for detecting active proliferative cells; vimentin was used for epithelial–mesenchymal transition (EMT, ab8978, Abcam, United Kingdom) in tumors; and ezrin was used for local invasion and metastasis potential (MA5-13862, ThermoFisher Scientific, United States). Imaging of H&E stain and immunohistochemistry marking was performed using a PANNORAMIC 250 Flash III Scanner (3DHISTECH, Budapest, Hungary). All images were digitally captured with the Panoramic Viewer/Case Viewer software (3DHISTECH), and semiquantitative analyses of Ki67, vimentin, and ezrin expression were performed using the Fiji open-source image processing program (ImageJ software, USA). H-scores, or histoscores, were obtained using a color deconvolution plugin (and with 20× magnification images), using the IHC Profiler Plugin developed by Varghese et al. [74]. For every TMA image—i.e., for every protein, patient, and passage—the H-score was calculated by applying the following formula [71,72,73]: (1 × % low-positive cells 1+) + (2 × % positive cells 2+) + (3 × % high-positive cells 3+).

### 4.6. Statistical Analyses

Survival analysis was performed by using the Mantel-Cox (logrank) test, and survival curves were estimated by the Kaplan-Meier method. Cumulative incidence curves for clinicopathological variables were also compared by logrank test. Regarding tumor growth in mice and variability between different median latency times (across passages and patients), these were evaluated by Friedman test, as not all samples passed normality tests. H-scores were analyzed by Spearman’s rank correlation coefficient, and correlation matrices were built, when the relationships between proteins were evaluated; and by the Mann-Whitney test when protein expression was compared with clinicopathological categorical and dichotomized variables, such as mutations, survival, age, stage, and histology. Other tests that were performed but did not show statistical significance in several types of comparison, were the Kruskal-Wallis, ANOVA, and Wilcoxon tests. These tests and analyses were developed using the scientific graphing and statistics software GraphPad Prism 8 (GraphPad Software, San Diego, CA, USA).

## 5. Conclusions

In conclusion, we successfully established nine PDXs of lung cancer patients, of which ADC tumors bearing the KRAS-G12C mutation were the most frequently engrafted. Our data indicate that the most aggressive tumors have a greater implantation capacity in mice and, therefore, the success of implantation in mice may be indicative of a poor prognosis. On the other hand, tumors in the PDXs—being in a different microenvironment, losing the stroma and the immune cells—can evolve during successive passages, and suffer a selective pressure towards more proliferative and mesenchymal cells, associated with higher levels of vimentin, ezrin, and Ki67 protein expression. Therefore, preclinical studies on PDXs may be performed preferentially in early passages, where the characteristics of the primary tumor and tumor microenvironment are still maintained. The high expression of vimentin, ezrin, and Ki67 proteins in tumors suggests greater aggressiveness and, therefore, the evaluation of their expression could be used in combination as a prognosis marker for the study of disease progression status. Moreover, Ki67, ezrin, and vimentin may serve as potential therapeutic targets in cancer. Thus, it is expected that identification of specific antisense nucleotides or small molecules to inhibit the activity of these proteins would lead to the discovery of antiproliferative and antimetastatic drugs [67,99,100]. This PDX collection provides a valuable platform for biomarker discovery and drug testing for preclinical studies.

## Figures and Tables

**Table 1:** Clinicopathological characteristics of the patients.

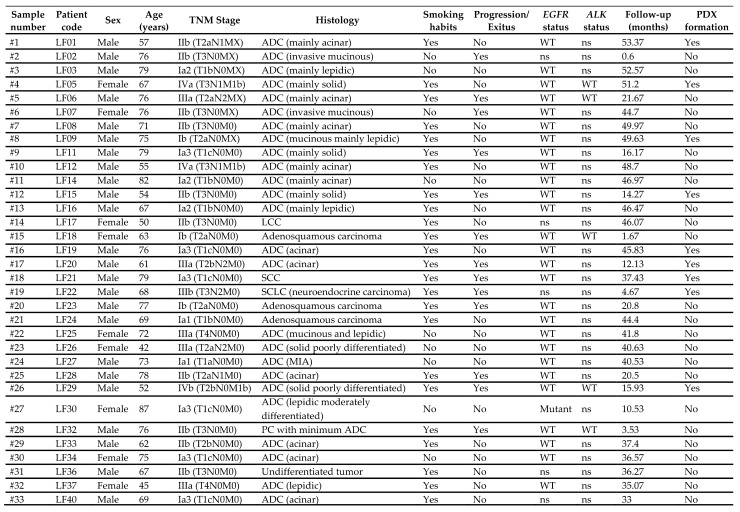

PDX: patient-derived xenograft; ADC: adenocarcinoma; SCC: squamous-cell carcinoma; LCC: large-cell lung carcinoma; SCLC: small-cell lung cancer; MIA: minimally invasive adenocarcinoma of the lung; PC: pulmonary pleomorphic carcinoma.

**Table 2:** PDX formation success according to different clinicopathological variables of the patients (*n* = 33).

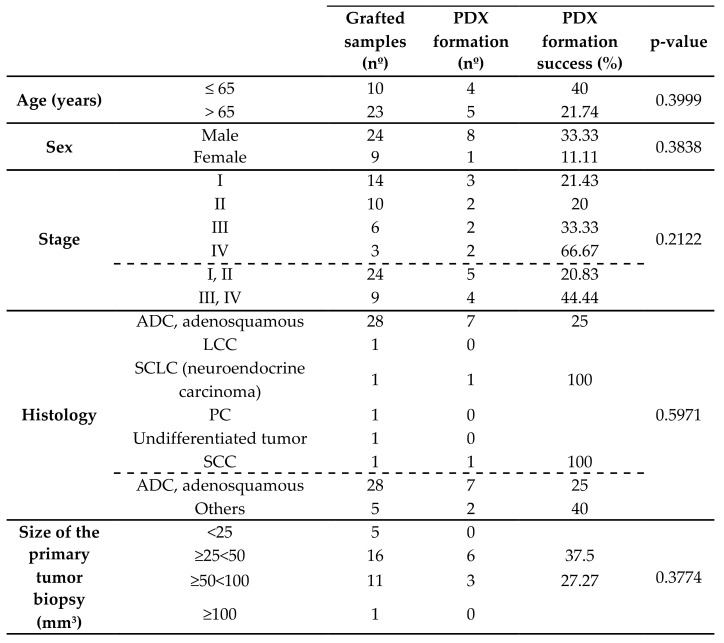

**Table 3:** Somatic mutation in surgically resected tumors that generated PDXs.

Sample Number	Patient Code	Genetic Alterations (Oncomine™ Focus Assay)	
#1	LF01	ERBB4 c.2139G>T; *p*.L713F	9% allele frequency
#4	LF05	KRAS c.34G>T; *p*.G12C	67% allele frequency
#8	LF09	KRAS c.34G>T; *p*.G12C	7% allele frequency
#12	LF15	KRAS c.34G>T; *p*.G12C ERBB2 c.2524G>A; *p*.V842I	32% allele frequency 4% allele frequency
#17	LF20	ERBB2 c.2301C>G; *p*.I767M MYC c.77A>G; *p*.N26S	74% allele frequency 58% allele frequency
#26	LF29	MET c.3029 C>T; *p*.T1010I	29% allele frequency

Samples from resected tumors that generated PDX were analyzed by massive sequencing with the Oncomine Focus Assay. The table shows the identified mutations.

## Data Availability

The authors confirm that the data supporting the conclusions reported in this study are available within the article and its Supplementary Materials which will be available in the public database PubMed. The authors also agree to provide the data upon request.

## Supplementary Materials

The following are available online at https://www.mdpi.com/2072-6694/13/12/2980/s1: Figure S1: Survival curves according to the clinicopathological characteristics of the patients; Figure S2: Flowchart of the in vivo PDX model generation; Figure S3: Friedman test statistics of MLT data; Figure S4: PDX tumor growth curves and median latency times; Figure S5: Tissue microarray panels with the immunohistochemical analyses per patient; Figure S6: Bar charts of H-Scores of Vimentin, Ezrin and Ki67 protein expression from the indicated primary tumor and PDX tumors during passages X0 to X2. Figure S7: H-score graphs of vimentin, ezrin, and Ki67 protein expression from all primary and PDX tumors; Table S1: H-scores of vimentin, ezrin, and Ki67 protein expression from primary tumors (PT) and PDX tumors.

## References

[1] American Cancer Society (2019). Cancer Facts & Figures 2019.

[2] International Agency for Research on Cancer (IARC) (2018). Latest global cancer data. http://www.who.int/cancer/PRGlobocanFinal.pdf.

[3] Langer C.J., Besse B., Gualberto A., Brambilla E., Soria J.-C. (2010). The evolving role of histology in the management of advanced non-small-cell lung cancer. J. Clin. Oncol..

[4] Chen Z., Fillmore C.M., Hammerman P.S., Kim C.F., Wong K.K. (2014). Non-small-cell lung cancers: A heterogeneous set of diseases. Nat. Rev. Cancer.

[5] Howlader N., Noone A.-M., Krapcho M., Miller D.K., Brest A., Yu M., Ruhl J., Tatalovich Z., Mariotto A., Lewis D. (2019). SEER Cancer Statistics Review, 1975–2016.

[6] Uramoto H., Tanaka F. (2014). Recurrence after surgery in patients with NSCLC. Transl. Lung Cancer Res..

[7] Yuan M., Huang L.-L., Chen J.-H., Wu J., Xu Q. (2019). The emerging treatment landscape of targeted therapy in non-small-cell lung cancer. Signal Transduct. Target Ther..

[8] Kang J., Zhang C., Zhong W.Z. (2021). Neoadjuvant immunotherapy for non-small cell lung cancer: State of the art. Cancer Commun..

[9] Bai R., Li L., Chen X., Chen N., Song W., Cui J. (2020). Neoadjuvant and Adjuvant Immunotherapy: Opening New Horizons for Patients With Early-Stage Non-small Cell Lung Cancer. Front Oncol..

[10] Hidalgo M., Amant F., Biankin A.V., Budinská E., Byrne A.T., Caldas C., Clarke R.B., Jong S.d., Jonkers J., Mælandsmo G.M. (2014). Patient-derived xenograft models: An emerging platform for translational cancer research. Cancer Discov..

[11] Kim M., Mun H., Sung C.O., Cho E.J., Jeon H.-J., Chun S.-M., Jung D.J., Shin T.H., Jeong G.S., Kim D.K. (2019). Patient-derived lung cancer organoids as in vitro cancer models for therapeutic screening. Nat. Commun..

[12] Sachs N., Clevers H. (2014). Organoid cultures for the analysis of cancer phenotypes. Curr. Opin. Genet. Dev..

[13] DeRose Y.S., Wang G., Lin Y.-C., Bernard P.S., Buys S.S., Ebbert M.T.W., Factor R., Matsen C., Milash B.A., Nelson E. (2011). Tumor grafts derived from women with breast cancer authentically reflect tumor pathology, growth, metastasis and disease outcomes. Nat. Med..

[14] Tentler J.J., Tan A.C., Weekes C.D., Jimeno A., Leong S., Pitts T.M., Arcaroli J.J., Messersmith W.A., Eckhardt S.G. (2012). Patient-derived tumour xenografts as models for oncology drug development. Nat. Rev. Clin. Oncol..

[15] Zhang Z., Wang H., Ding Q., Xing Y., Xu Z., Lu C., Luo D., Xu L., Xia W., Zhou C. (2018). Establishment of patient-derived tumor spheroids for non-small cell lung cancer. PLoS One.

[16] Dobrolecki L.E., Airhart S.D., Alferez D.G., Aparicio S., Behbod F., Bentires-Alj M., Brisken C., Bult C.J., Cai S., Clarke R.B. (2016). Patient-derived xenograft (PDX) models in basic and translational breast cancer research. Cancer Metastasis Rev..

[17] Kalluri R., Weinberg R.A. (2009). The basics of epithelial-mesenchymal transition. J. Clin. Investig..

[18] Thiery J.P., Acloque H., Huang R.Y.J., Nieto M.A. (2009). Epithelial-Mesenchymal Transitions in Development and Disease. Cell.

[19] Nieto M.A., Huang R.Y.Y.J., Jackson R.A.A., Thiery J.P.P. (2016). EMT: 2016. Cell.

[20] Yu M., Bardia A., Wittner B.S., Stott S.L., Smas M.E., Ting D.T., Isakoff S.J., Ciciliano J.C., Wells M.N., Shah A.M. (2013). Circulating breast tumor cells exhibit dynamic changes in epithelial and mesenchymal composition. Science.

[21] Huang R.Y.-J., Wong M.K., Tan T.Z., Kuay K.T., Ng A.H.C., Chung V.Y., Chu Y.-S., Matsumura N., Lai H.-C., Lee Y.F. (2013). An EMT spectrum defines an anoikis-resistant and spheroidogenic intermediate mesenchymal state that is sensitive to e-cadherin restoration by a src-kinase inhibitor, saracatinib (AZD0530). Cell Death Dis..

[22] Jordan N.V., Johnson G.L., Abell A.N. (2011). Tracking the intermediate stages of epithelial-mesenchymal transition in epithelial stem cells and cancer. Cell Cycle.

[23] Zheng X., Carstens J.L., Kim J., Scheible M., Kaye J., Sugimoto H., Wu C.-C., LeBleu V.S., Kalluri R. (2015). Epithelial-to-mesenchymal transition is dispensable for metastasis but induces chemoresistance in pancreatic cancer. Nature.

[24] Diepenbruck M., Christofori G. (2016). Epithelial-mesenchymal transition (EMT) and metastasis: Yes, no, maybe?. Curr. Opin. Cell Biol..

[25] Ocaña O.H., Córcoles R., Fabra A., Moreno-Bueno G., Acloque H., Vega S., Barrallo-Gimeno A., Cano A., Nieto M.A. (2012). Metastatic colonization requires the repression of the epithelial-mesenchymal transition inducer Prrx1. Cancer Cell.

[26] Nieto M.A. (2013). Epithelial plasticity: A common theme in embryonic and cancer cells. Science.

[27] Wellner U., Schubert J., Burk U.C., Schmalhofer O., Zhu F., Sonntag A., Waldvogel B., Vannier C., Darling D., zur Hausen A. (2009). The EMT-activator ZEB1 promotes tumorigenicity by repressing stemness-inhibiting microRNAs. Nat. Cell Biol..

[28] Plaks V., Kong N., Werb Z. (2015). The cancer stem cell niche: How essential is the niche in regulating stemness of tumor cells?. Cell Stem Cell.

[29] Singh A., Settleman J. (2010). EMT, cancer stem cells and drug resistance: An emerging axis of evil in the war on cancer. Oncogene.

[30] Vega S., Morales A.V., Ocaña O.H., Valdés F., Fabregat I., Nieto M.A. (2004). Snail blocks the cell cycle and confers resistance to cell death. Genes Dev..

[31] Rana M.K., Aloisio F.M., Choi C., Barber D.L. (2018). Formin-dependent TGF-β signaling for epithelial to mesenchymal transition. Mol. Biol. Cell..

[32] Lamouille S., Xu J., Derynck R. (2014). Molecular mechanisms of epithelial—mesenchymal transition. Nat. Rev. Mol. Cell Biol..

[33] Thiery J.P., Sleeman J.P. (2006). Complex networks orchestrate epithelial-mesenchymal transitions. Nat. Rev. Mol. Cell Biol..

[34] Yilmaz M., Christofori G. (2009). EMT, the cytoskeleton, and cancer cell invasion. Cancer Metastasis Rev..

[35] Mendez M.G., Kojima S.-I., Goldman R.D. (2010). Vimentin induces changes in cell shape, motility, and adhesion during the epithelial to mesenchymal transition. FASEB J. Off. Publ. Fed. Am. Soc. Exp. Biol..

[36] Chesarone M.A., DuPage A.G., Goode B.L. (2010). Unleashing formins to remodel the actin and microtubule cytoskeletons. Nat. Rev. Mol. Cell Biol..

[37] Clucas J., Valderrama F. (2015). ERM proteins in cancer progression. J. Cell Sci..

[38] Chen M.J., Gao X.J., Xu L.N., Liu T.F., Liu X.H., Liu L.X. (2014). Ezrin is required for epithelial-mesenchymal transition induced by TGF-β1 in A549 cells. Int. J. Oncol..

[39] Li Q., Gao H., Xu H., Wang X., Pan Y., Hao F., Qiu X., Stoecker M., Wang E., Wang E. (2012). Expression of ezrin correlates with malignant phenotype of lung cancer, and in vitro knockdown of ezrin reverses the aggressive biological behavior of lung cancer cells. Tumour Biol. J. Int. Soc. Oncodevelopmental Biol. Med..

[40] Jiang Y., Zhao J., Zhang Y., Li K., Li T., Chen X., Zhao S., Zhao S., Liu K., Dong Z. (2018). Establishment of lung cancer patient-derived xenograft models and primary cell lines for lung cancer study. J. Transl. Med..

[41] Moro M., Bertolini G., Caserini R., Borzi C., Boeri M., Fabbri A., Leone G., Gasparini P., Galeone C., Pelosi G. (2017). Establishment of patient derived xenografts as functional testing of lung cancer aggressiveness. Sci. Rep..

[42] Lee J.W., Soung Y.H., Seo S.H., Kim S.Y., Park C.H., Wang Y.P., Park K., Nam S.W., Park W.S., Kim S.H. (2006). Somatic mutations of ERBB2 kinase domain in gastric, colorectal, and breast carcinomas. Clin. Cancer Res. Off. J. Am. Assoc. Cancer Res..

[43] Grenda A., Krawczyk P., Chmielewska I., Nicoś M., Milanowski J. (2020). Questions around mutation T1010I in MET gene: Results of next generation sequencing in Polish patient with suspected hereditary adenoid cystic carcinoma. Eur. Rev. Med. Pharmacol. Sci..

[44] Ma P.C., Kijima T., Maulik G., Fox E.A., Sattler M., Griffin J.D., Johnson B.E., Salgia R. (2003). c-MET mutational analysis in small cell lung cancer: Novel juxtamembrane domain mutations regulating cytoskeletal functions. Cancer Res..

[45] Johnston J.J., Rubinstein W.S., Facio F.M., Ng D., Singh L.N., Teer J.K., Mullikin J.C., Biesecker L.G. (2012). Secondary variants in individuals undergoing exome sequencing: Screening of 572 individuals identifies high-penetrance mutations in cancer-susceptibility genes. Am. J. Hum. Genet..

[46] Bean L.J.H., Tinker S.W., da Silva C., Hegde M.R. (2013). Free the data: One laboratory’s approach to knowledge-based genomic variant classification and preparation for EMR integration of genomic data. Hum. Mutat..

[47] Tate J.G., Bamford S., Jubb H.C., Sondka Z., Beare D.M., Bindal N., Boutselakis H., Cole C.G., Creatore C., Dawson E. (2019). COSMIC: The Catalogue Of Somatic Mutations In Cancer. Nucleic Acids Res..

[48] Shah S.P., Morin R.D., Khattra J., Prentice L., Pugh T., Burleigh A., Delaney A., Gelmon K., Guliany R., Senz J. (2009). Mutational evolution in a lobular breast tumour profiled at single nucleotide resolution. Nature.

[49] Deniziaut G., Tille J.C., Bidard F.-C., Vacher S., Schnitzler A., Chemlali W., Trémoulet L., Fuhrmann L., Cottu P., Rouzier R. (2016). ERBB2 mutations associated with solid variant of high-grade invasive lobular breast carcinomas. Oncotarget.

[50] Clark H.M., Yano T., Otsuki T., Jaffe E.S., Shibata D., Raffeld M. (1994). Mutations in the coding region of c-MYC in AIDS-associated and other aggressive lymphomas. Cancer Res..

[51] Mateo J., Seed G., Bertan C., Rescigno P., Dolling D., Figueiredo I., Miranda S., Nava Rodrigues D., Gurel B., Clarke M. (2020). Genomics of lethal prostate cancer at diagnosis and castration resistance. J. Clin. Invest..

[52] Gibert J., Clavé S., Hardy-Werbin M., Taus Á., Rocha P., Longarón R., Piquer G., Chaib I., Carcereny E., Morán T. (2020). Concomitant genomic alterations in KRAS mutant advanced lung adenocarcinoma. Lung Cancer..

[53] Ilie M., Nunes M., Blot L., Hofman V., Long-Mira E., Butori C., Selva E., Merino-Trigo A., Vénissac N., Mouroux J. (2015). Setting up a wide panel of patient-derived tumor xenografts of non-small cell lung cancer by improving the preanalytical steps. Cancer Med..

[54] Hao C., Wang L., Peng S., Cao M., Li H., Hu J., Huang X., Liu W., Zhang H., Wu S. (2015). Gene mutations in primary tumors and corresponding patient-derived xenografts derived from non-small cell lung cancer. Cancer Lett..

[55] Fichtner I., Rolff J., Soong R., Hoffmann J., Hammer S., Sommer A., Becker M., Merk J. (2008). Establishment of patient-derived non-small cell lung cancer xenografts as models for the identification of predictive biomarkers. Clin. Cancer Res. Off. J. Am. Assoc. Cancer Res..

[56] Lee H.W., Lee J.-I., Lee S.J., Cho H.J., Song H.J., Jeong D.E., Seo Y.J., Shin S., Joung J.-G., Kwon Y.-J. (2015). Patient-derived xenografts from non-small cell lung cancer brain metastases are valuable translational platforms for the development of personalized targeted therapy. Clin. cancer Res. an Off. J. Am. Assoc. Cancer Res..

[57] Cuenca R.E., Takita H., Bankert R. (1996). Orthotopic engraftment of human lung tumours in SCID mice for the study of metastasis. Surg. Oncol..

[58] Russo M.V., Faversani A., Gatti S., Ricca D., Del Gobbo A., Ferrero S., Palleschi A., Vaira V., Bosari S. (2015). A new mouse avatar model of non-small cell lung cancer. Front. Oncol..

[59] Moldvay J., Jackel M., Bogos K., Soltész I., Agócs L., Kovács G., Schaff Z. (2004). The role of TTF-1 in differentiating primary and metastatic lung adenocarcinomas. Pathol. Oncol. Res..

[60] Bingle C.D. (1997). Thyroid transcription factor-1. Int. J. Biochem. Cell Biol..

[61] Byrne A.T., Alférez D.G., Amant F., Annibali D., Arribas J., Biankin A.V., Bruna A., Budinská E., Caldas C., Chang D.K. (2017). Interrogating open issues in cancer precision medicine with patient-derived xenografts. Nat. Rev. Cancer.

[62] Whittle J.R., Lewis M.T., Lindeman G.J., Visvader J.E. (2015). Patient-derived xenograft models of breast cancer and their predictive power. Breast. Cancer Res..

[63] Dauphin M., Barbe C., Lemaire S., Nawrocki-Raby B., Lagonotte E., Delepine G., Birembaut P., Gilles C., Polette M. (2013). Vimentin expression predicts the occurrence of metastases in non small cell lung carcinomas. Lung Cancer.

[64] Battaglia R.A., Delic S., Herrmann H., Snider N.T. (2018). Vimentin on the move: New developments in cell migration. F1000Research.

[65] Liu S., Liu L., Ye W., Ye D., Wang T., Guo W., Liao Y., Xu D., Song H., Zhang L. (2016). High Vimentin Expression Associated with Lymph Node Metastasis and Predicated a Poor Prognosis in Oral Squamous Cell Carcinoma. Sci. Rep..

[66] Moodley  S., Lian E.Y., Crupi  M.J.F., Hyndman B.D., Mulligan L.M. (2020). RET isoform-specific interaction with scaffold protein Ezrin promotes cell migration and chemotaxis in lung adenocarcinoma. Lung Cancer..

[67] Song Y., Ma X., Zhang M., Wang M., Wang G., Ye Y., Xia W. (2020). Ezrin Mediates Invasion and Metastasis in Tumorigenesis: A Review. Front. Cell Dev. Biol..

[68] Gerdes J. (1990). Ki-67 and other proliferation markers useful for immunohistological diagnostic and prognostic evaluations in human malignancies. Semin. Cancer Biol..

[69] Martin B., Paesmans M., Mascaux C., Berghmans T., Lothaire P., Meert A.-P., Lafitte J.-J., Sculier J.-P. (2004). Ki-67 expression and patients survival in lung cancer: Systematic review of the literature with meta-analysis. Br. J. Cancer.

[70] Chirieac L.R. (2016). Ki-67 expression in pulmonary tumors. Transl. Lung Cancer Res..

[71] Varghese F., Bukhari A.B., Malhotra R., De A. (2014). IHC profiler: An open source plugin for the quantitative evaluation and automated scoring of immunohistochemistry images of human tissue samples. PLoS ONE.

[72] Ishibashi H., Suzuki T., Suzuki S., Moriya T., Kaneko C., Sasano H. (2003). Sex Steroid Hormone Receptors in Human Thymoma. J. Clin. Endocrinol. Metab..

[73] Koopman L.A., Terp M.G., Zom G.G., Janmaat M.L., Jacobsen K., Van Den Heuvel E.G., Brandhorst M., Forssmann U., De Bree F., Pencheva N. (2019). Enapotamab vedotin, an AXL-specific antibody-drug conjugate, shows preclinical antitumor activity in non-small cell lung cancer. JCI Insight.

[74] Hirsch F.R., Varella-Garcia M., Bunn P.A.J., Di Maria M.V., Veve R., Bremmes R.M., Barón A.E., Zeng C., Franklin W.A. (2003). Epidermal growth factor receptor in non-small-cell lung carcinomas: Correlation between gene copy number and protein expression and impact on prognosis. J. Clin. Oncol. Off. J. Am. Soc. Clin. Oncol..

[75] Madan R., Brandwein-Gensler M., Schlecht N.F., Elias K., Gorbovitsky E., Belbin T.J., Mahmood R., Breining D., Qian H., Childs G. (2006). Differential tissue and subcellular expressionof ERM proteins in normal and malignant tissues: Cytoplasmic ezrin expression has prognostic signficance for head and neck squamous cell carcinoma. Head Neck.

[76] Shibue T., Weinberg R.A. (2017). EMT, CSCs, and drug resistance: The mechanistic link and clinical implications. Nat. Rev. Clin. Oncol..

[77] Woo T., Okudela K., Yazawa T., Wada N., Ogawa N., Ishiwa N., Tajiri M., Rino Y., Kitamura H., Masuda M. (2009). Prognostic value of KRAS mutations and Ki-67 expression in stage I lung adenocarcinomas. Lung Cancer.

[78] Morgan K.M., Riedlinger G.M., Rosenfeld J., Ganesan S., Pine S.R. (2017). Patient-derived xenograft models of non-small cell lung cancer and their potential utility in personalized medicine. Front. Oncol..

[79] Guenot D., Guérin E., Aguillon-Romain S., Pencreach E., Schneider A., Neuville A., Chenard M.-P., Duluc I., Du Manoir S., Brigand C. (2006). Primary tumour genetic alterations and intra-tumoral heterogeneity are maintained in xenografts of human colon cancers showing chromosome instability. J. Pathol..

[80] Kita K., Fukuda K., Takahashi H., Tanimoto A., Nishiyama A., Arai S., Takeuchi S., Yamashita K., Ohtsubo K., Otani S. (2019). Patient-derived xenograft models of non-small cell lung cancer for evaluating targeted drug sensitivity and resistance. Cancer Sci..

[81] Baschnagel A.M., Kaushik S., Durmaz A., Goldstein S., Ong I.M., Abel L., Clark P.A., Gurel Z., Leal T., Buehler D. (2021). Development and characterization of patient-derived xenografts from non-small cell lung cancer brain metastases. Sci. Rep..

[82] Ben-David U., Ha G., Tseng Y.Y., Greenwald N.F., Oh C., Shih J., McFarland J.M., Wong B., Boehm J.S., Beroukhim R. (2017). Patient-derived xenografts undergo mouse-specific tumor evolution. Nat. Genet..

[83] Ben-David U., Beroukhim R., Golub T.R. (2019). Genomic evolution of cancer models: Perils and opportunities. Nat. Rev. Cancer..

[84] Moore A.R., Rosenberg S.C., McCormick F., Malek S. (2020). RAS-targeted therapies: Is the undruggable drugged?. Nat. Rev. Drug. Discov..

[85] Hugo H., Ackland M.L., Blick T., Lawrence M.G., Clements J.A., Williams E.D., Thompson E.W. (2007). Epithelial--mesenchymal and mesenchymal--epithelial transitions in carcinoma progression. J. Cell. Physiol..

[86] Ye Z., Zhang X., Luo Y., Li S., Huang L., Li Z., Li P., Chen G. (2016). Prognostic Values of Vimentin Expression and Its Clinicopathological Significance in Non-Small Cell Lung Cancer: A Meta-Analysis of Observational Studies with 4118 Cases. PLoS One.

[87] Zhang X., Li G., Guo Y., Song Y., Chen L., Ruan Q., Wang Y., Sun L., Hu Y., Zhou J. (2019). Regulation of ezrin tension by S-nitrosylation mediates non-small cell lung cancer invasion and metastasis. Theranostics.

[88] Lee H.W., Kim E.H., Oh M.-H. (2012). Clinicopathologic implication of ezrin expression in non-small cell lung cancer. Korean J. Pathol..

[89] Jin T., Jin J., Li X., Zhang S., Choi Y.H., Piao Y., Shen X., Lin Z. (2014). Prognostic implications of ezrin and phosphorylated ezrin expression in non-small cell lung cancer. BMC Cancer.

[90] Zacharias M., Brcic L., Eidenhammer S., Popper H. (2018). Bulk tumour cell migration in lung carcinomas might be more common than epithelial-mesenchymal transition and be differently regulated. BMC Cancer.

[91] Zhang Y., Wang L.F., Gao J.H., Li L., Jiang P., Lv X., Yu L.X., Yang J., Li R.T., Liu B.R. (2019). Clinical significance of epithelial-mesenchymal transition-related molecules in lung adenocarcinoma. Curr. Oncol..

[92] Matsubara T., Tagawa T., Takada K., Toyokawa G., Shimokawa M., Kozuma Y., Akamine T., Haro A., Osoegawa A., Mori M. (2019). Clinical and Prognostic Significance of the Epithelial-Mesenchymal Transition in Stage IA Lung Adenocarcinoma: A Propensity Score-Matched Analysis. Clin. Lung Cancer.

[93] Karnoub A.E., Weinberg R.A. (2008). Ras oncogenes: Split personalities. Nat. Rev. Mol. Cell Biol..

[94] Richardson A.M., Havel L.S., Koyen A.E., Konen J.M., Shupe J., Wiles 4th W.G., Martin W.D., Grossniklaus H.E., Sica G., Gilbert-Ross M. (2018). Vimentin Is Required for Lung Adenocarcinoma Metastasis via Heterotypic Tumor Cell-Cancer-Associated Fibroblast Interactions during Collective Invasion. Clin. Cancer Res. Off. J. Am. Assoc. Cancer Res..

[95] Yin L.-M., Duan T.-T., Ulloa L., Yang Y.-Q. (2018). Ezrin Orchestrates Signal Transduction in Airway Cells. Rev. Physiol. Biochem. Pharmacol..

[96] Slik K., Kurki S., Korpela T., Carpén O., Korkeila E., Sundström J. (2017). Ezrin expression combined with MSI status in prognostication of stage II colorectal cancer. PLoS ONE.

[97] Saygideğer-Kont Y., Minas T.Z., Jones H. (2016). Ezrin Enhances EGFR Signaling and Modulates Erlotinib Sensitivity in Non-Small Cell Lung Cancer Cells. Neoplasia.

[98] Parsons M., Grabsch H. (2009). How to make tissue microarrays. Diagnostic. Histopathol..

[99] Yang C., Zhang J., Ding M., Xu K., Li L., Mao L., Zheng J. (2018). Ki67 targeted strategies for cancer therapy. Clin. Transl. Oncol. Off. Publ. Fed. Spanish Oncol. Soc. Natl. Cancer Inst. Mex..

[100] Strouhalova K., Přechová M., Gandalovičová A., Brábek J., Gregor M., Rosel D. (2020). Vimentin Intermediate Filaments as Potential Target for Cancer Treatment. Cancers.

