# Correction: Pardo-Sánchez et al. Increased Tumor Growth Rate and Mesenchymal Properties of NSCLC-Patient-Derived Xenograft Models during Serial Transplantation. *Cancers* 2021, *13*, 2980

**DOI:** 10.3390/cancers13194825

**Published:** 2021-09-27

**Authors:** José Miguel Pardo-Sánchez, Nuria Mancheño, José Cerón, Carlos Jordá, Emilio Ansotegui, Óscar Juan, Sarai Palanca, Antonio Cremades, Carolina Gandía, Rosa Farràs

**Affiliations:** 1 Oncogenic Signalling Laboratory, Centro de Investigación Príncipe Felipe, 46012 Valencia, Spain; jmpardo@cipf.es (J.M.P.-S.); cgandia@cipf.es (C.G.); 2 Department of Pathology, University and Polytechnic La Fe Hospital, 46026 Valencia, Spain; manchenyo_nur@gva.es; 3 Department of Thoracic Surgery, University and Polytechnic La Fe Hospital, 46026 Valencia, Spain; ceron_jos@gva.es (J.C.); jorda_car@gva.es (C.J.); 4 Department of Pulmonology, University and Polytechnic La Fe Hospital, 46026 Valencia, Spain; ansotegui_emi@gva.es; 5 Department of Medical Oncology, University and Polytechnic La Fe Hospital, 46026 Valencia, Spain; juan_osc@gva.es; 6 Molecular Biology Unit, Service of Clinical Analysis, University and Polytechnic La Fe Hospital, 46026 Valencia, Spain; palanca_sar@gva.es; 7 Department of Pathology, Hospital Universitario de la Ribera, 46600 Alzira, Spain; cremades_antmir@gva.es

The authors would like to make a correction to their published paper [1].

There was a mistake in the original version of the article.

1. Figure S1A was uploaded instead of Figure 2A. Thus, Figure 2 should be replaced with the following version:

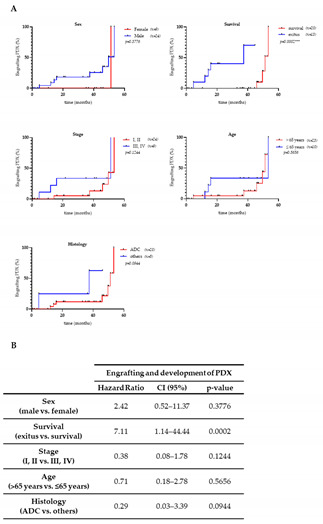

2. In Table 3, the % allele frequencies of samples #17 and 26# were missing. The original Table 3 should be replaced with the following Table 3:
3. In the Funding Section, acknowledgment to co-funding by ERDF/ESF, “Investing in your future” was missing. The Funding Section should be replaced with the following statement:

**Funding:** This research was funded by the Fondo de Investigación Sanitaria, ISCIII, grant numbers PI15-209 and PI20-194, co-funded by ERDF/ESF, “Investing in your future”. J.M.P.-S. was funded by the Ministerio de Educación, Cultura, y Deporte, grant number FPU13/02755.

4. In addition, some minor typos have been corrected.

In the paragraph after Figure 2, “p.N11S” should be replaced with “p.N26S”.

In the paragraph before the subheading of Section 2.5, “increased” should be replaced with “increase”.

In the second paragraph of the Discussion Section, “SLCL” should be replaced with “SCLC”.

In the penultimate paragraph of the Discussion Section, “NSLCL” should be replaced with “NSCLC”.

The authors apologize for any inconvenience caused and state that the scientific conclusions are unaffected. The original article has been updated.

## Figures and Tables

**Table 3:** Somatic mutation in surgically resected tumors that generated PDXs.

Sample Number	Patient Code	Genetic Alterations (Oncomine™ Focus Assay)	
#1	LF01	ERBB4 c.2139G>T; *p*.L713F	9% allele frequency
#4	LF05	KRAS c.34G>T; *p*.G12C	67% allele frequency
#8	LF09	KRAS c.34G>T; *p*.G12C	7% allele frequency
#12	LF15	KRAS c.34G>T; *p*.G12C ERBB2 c.2524G>A; *p*.V842I	32% allele frequency 4% allele frequency
#17	LF20	ERBB2 c.2301C>G; *p*.I767M MYC c.77A>G; *p*.N26S	74% allele frequency 58% allele frequency
#26	LF29	MET c.3029 C>T; *p*.T1010I	29% allele frequency
