# Supplementary material for: Increased Tumor Growth Rate and Mesenchymal Properties of NSCLC-Patient-Derived Xenograft Models during Serial Transplantation
Source: Cancers (Basel). 2021 Jun 14;13(12):2980. doi: 10.3390/cancers13122980 (PMC8232339; doi:10.3390/cancers13122980)
Supplement: Supplementary file 1 [file cancers-13-02980-s001.zip › cancers-1180453-supplementary.pdf]

Supplementary Materials: Increased tumor growth rate and mesenchymal properties of patient-derived xenograft models of NSCLC during serial transplantation

José Miguel Pardo-Sánchez, Nuria Mancheño, José Cerón, Carlos Jordá, Emilio Ansótegui, Óscar Juan, Sarai Palanca, Antonio Cremades, Carolina Gandía and Rosa Farràs

A

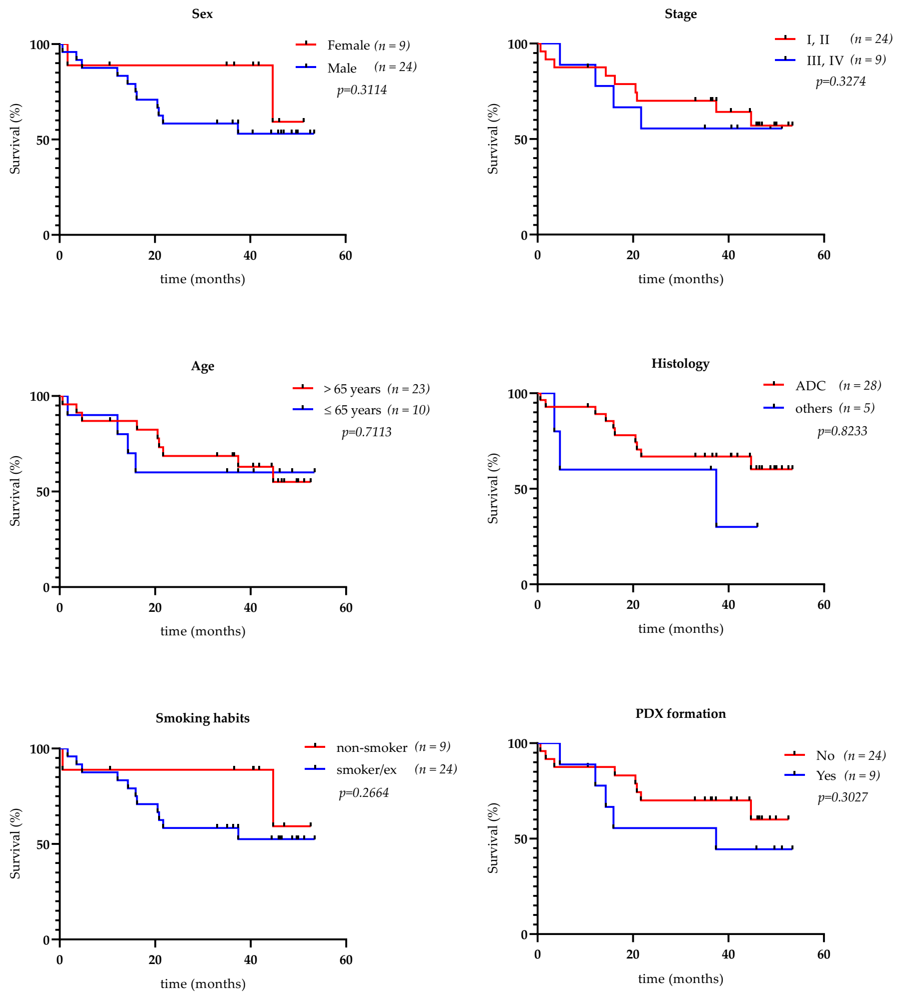

B

	Survival		
	**Hazard Ratio**	**CI (95%)**	**p-value**
**Sex (male vs. female)**	2.14	0.63–7.17	0.3114
**Stage (I, II vs. III, IV)**	0.80	0.23–2.76	0.3274
**Age (>65 years vs. ≤65 years)**	0.88	0.26-2.94	0.7113
**Histology (ADC vs. others)**	0.49	0.1-2.49	0.8233
**Smoking habits (smoker vs. non-smoker)**	2.16	0.65-7.23	0.2664
**PDX formation (yes vs. no)**	1.73	0.51-5.92	0.3027

Figure SF1. Survival curves according to the clinicopathological characteristics of the patients. (A) Kaplan-meier curves. (B) Log-rank test results (Mantel-Cox) correlating survival and the different clinicopathological characteristic of the patients. Hazard ratio, confidence intervals (95%) and p-value are shown. PDX: patient-derived xenograft. ADC: adenocarcinoma. CI: confidence Interval.

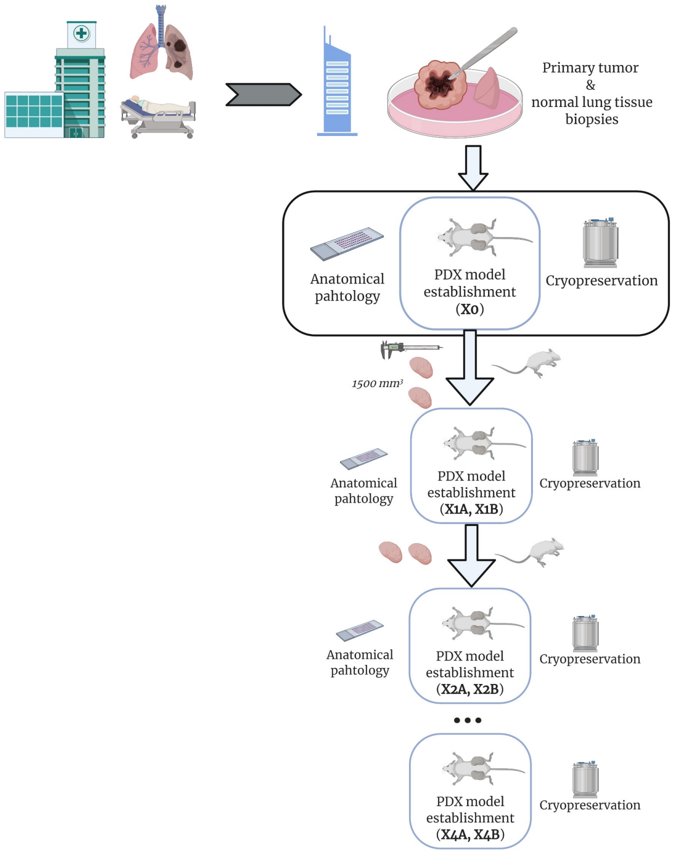

Figure SF2. Flowchart of the *in vivo* PDX model generation. A fragment of tumor sample (5mm^3) from lung cancer patient are implanted in the flank of 1 NOD scid gamma (NSG) immunocompromised mice (X0). When the tumor reaches around 1500 mm^3 size it is removed from donor mice, fragmented to 5 mm^3 portions, and two fragments (X1A, X1B) are serially transplanted in a new recipient mouse to establish subsequent PDX models. Portions of the PDX tumors are stored for histological analysis or cryopreserved for further studies. *PDX: patient-derived xenografts. TMA: tissue microarray. Created with BioRender.com*

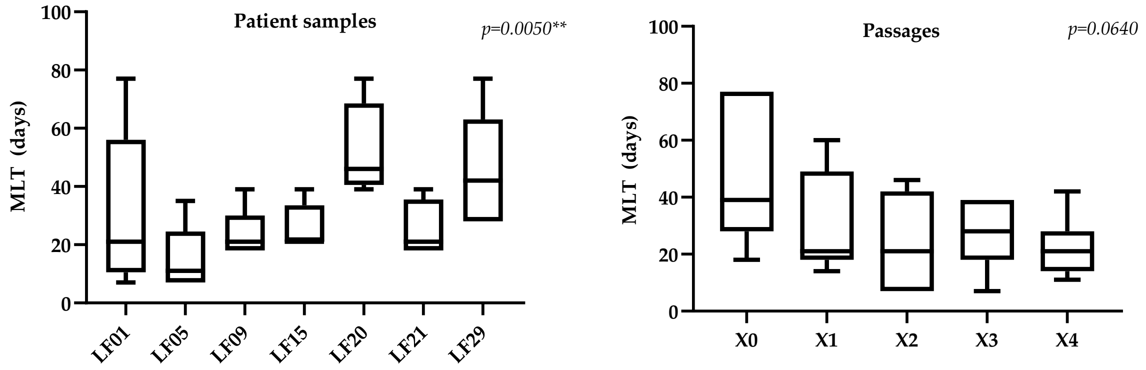

Figure SF3. Friedman test statistics of MLT data. (Left panel) Friedman test statistics of MLT data of all samples except LF19. (Right Panel) Friedman statistic test statistics of MLT data of all passages except LF19.

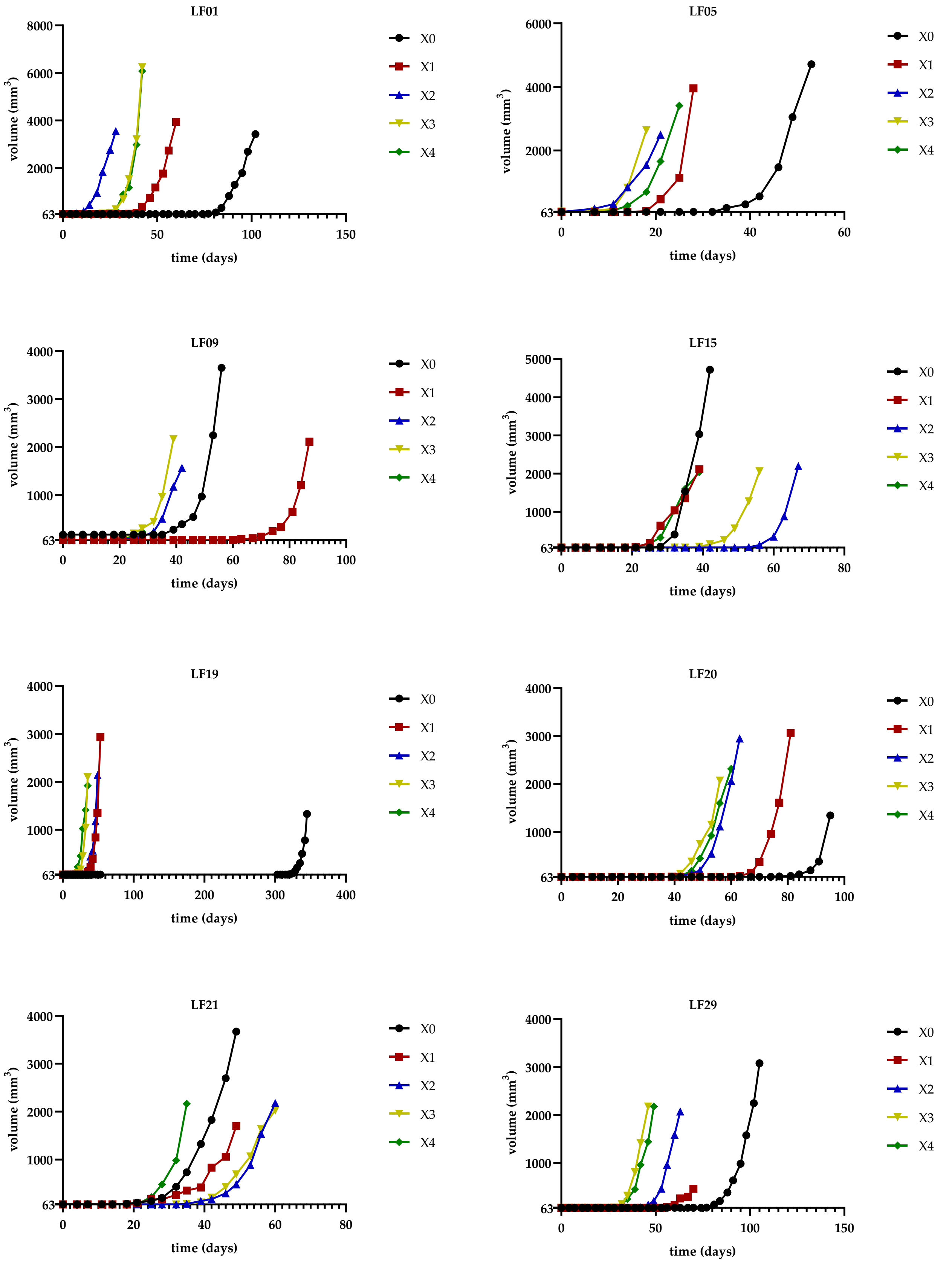

Figure SF4. PDX tumor growth curves and median latency times. Growth curves of PDX tumor replicas of passages X0 to X4 from each sample are represented as volume (mm^3) per time (days). The primary tumor implantation in mice is X0 and the last PDX tumor passage is X4.

Figure SF5. Tissue microarray panels with the immunohistochemical analyses per patient. Hematoxylin and eosine staining, and descriptive markers (CK7, CK20, TTF1) immnuhistochemistry are shown in panel A, while Vimentin, Ezrin and Ki67 immnuhistochemistry are shown in panel B, for each sample. Different magnifications are indicated: 5X, 20X, 40X. Panels for LF01, LF05, LF09, LF15, LF19, LF20, LF21, LF29 are shown.

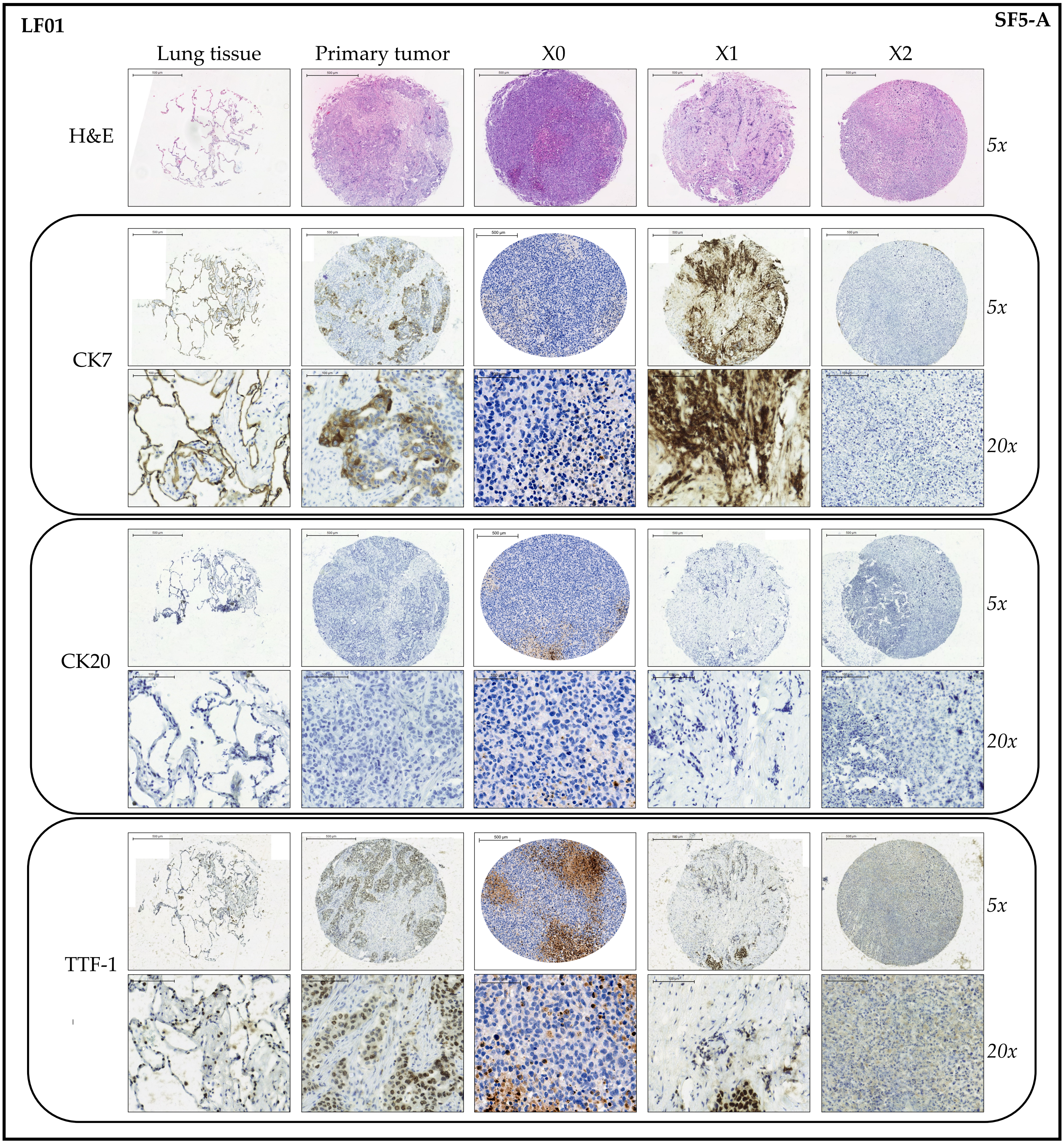

A) LF01: descriptive panel.

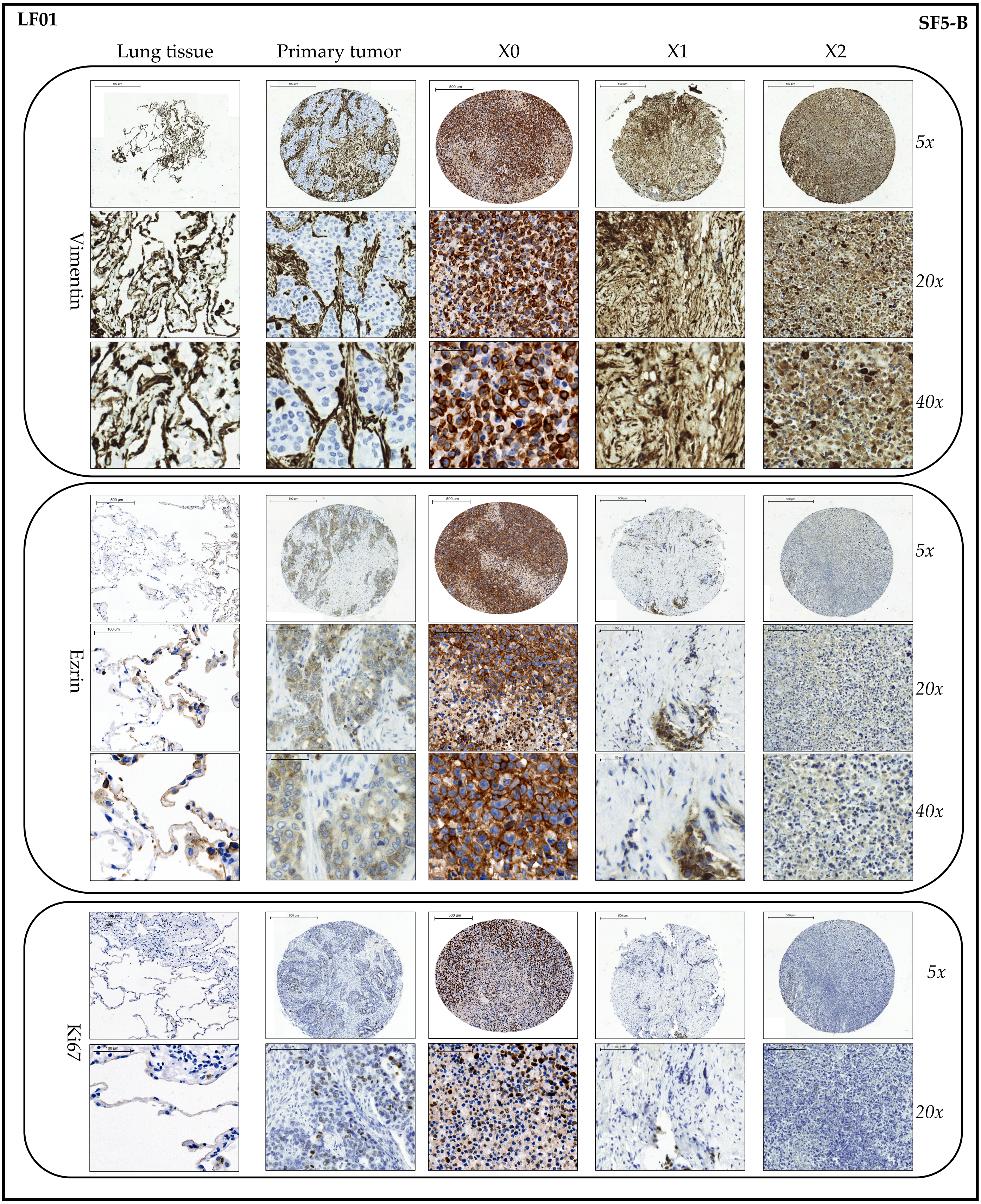

B) LF01: Vimentin, Ezrin and Ki67 panel.

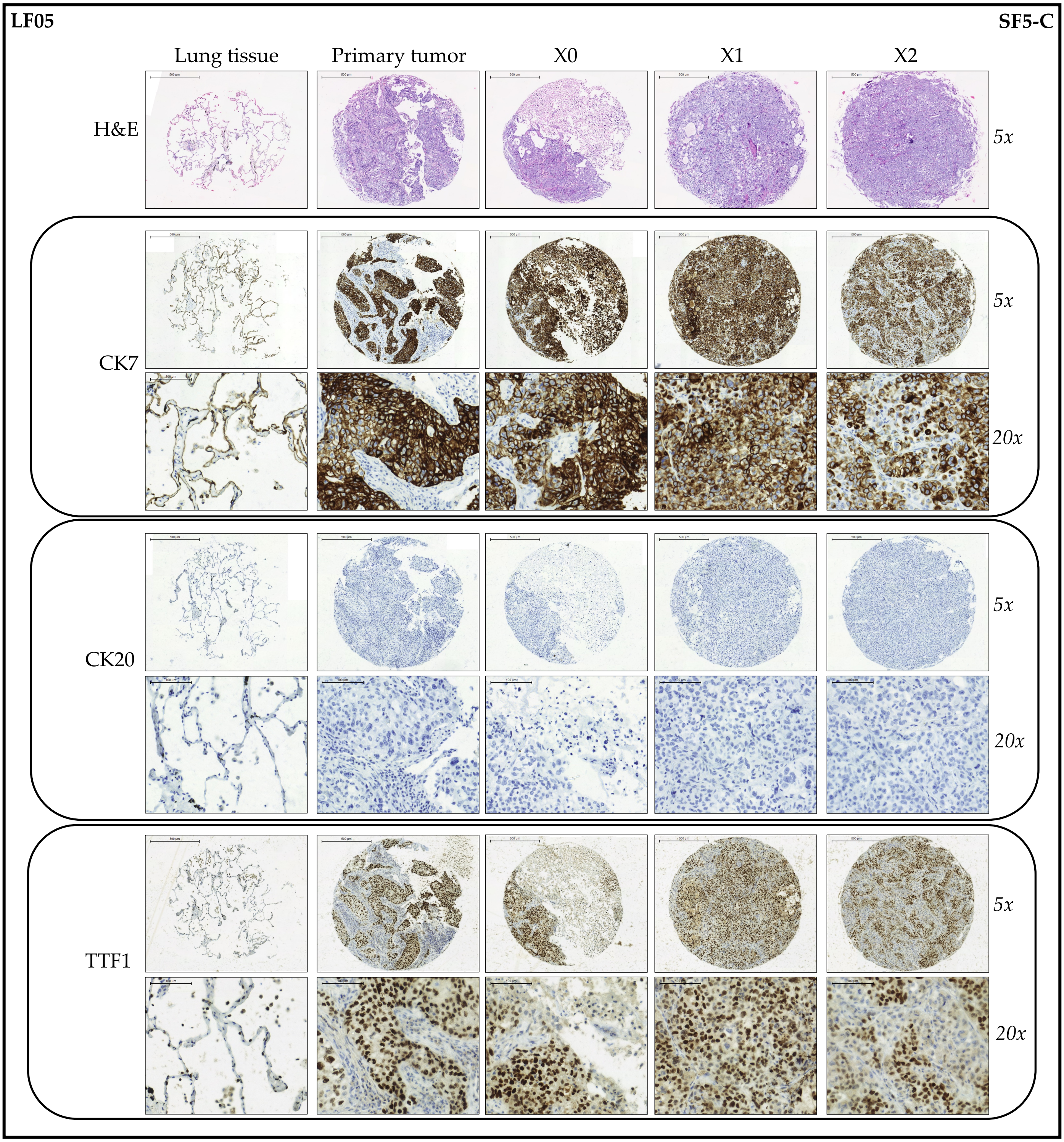

C) LF05: descriptive panel.

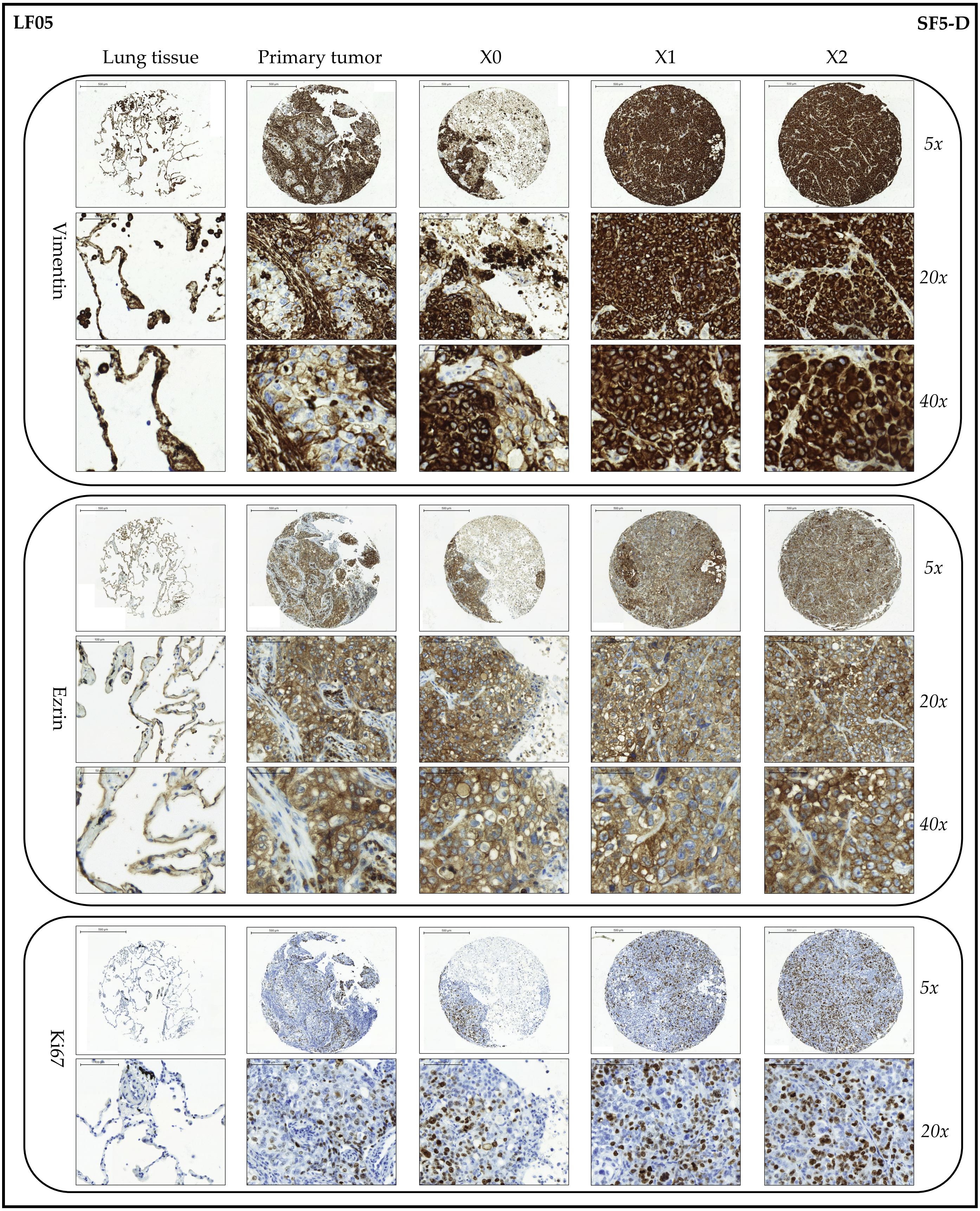

D) LF05: Vimentin, Ezrin and Ki67 panel.

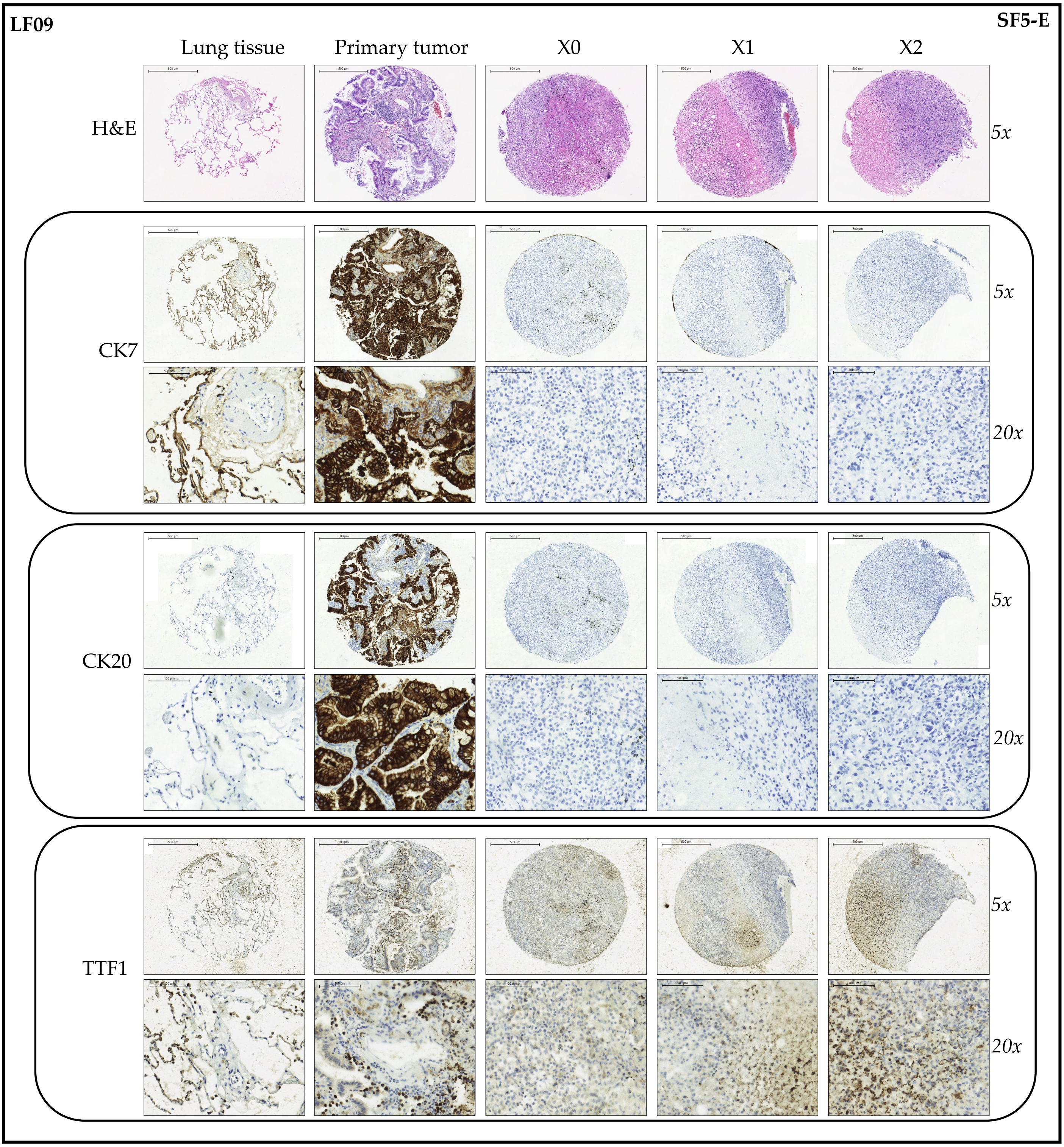

E) LF09: descriptive panel.

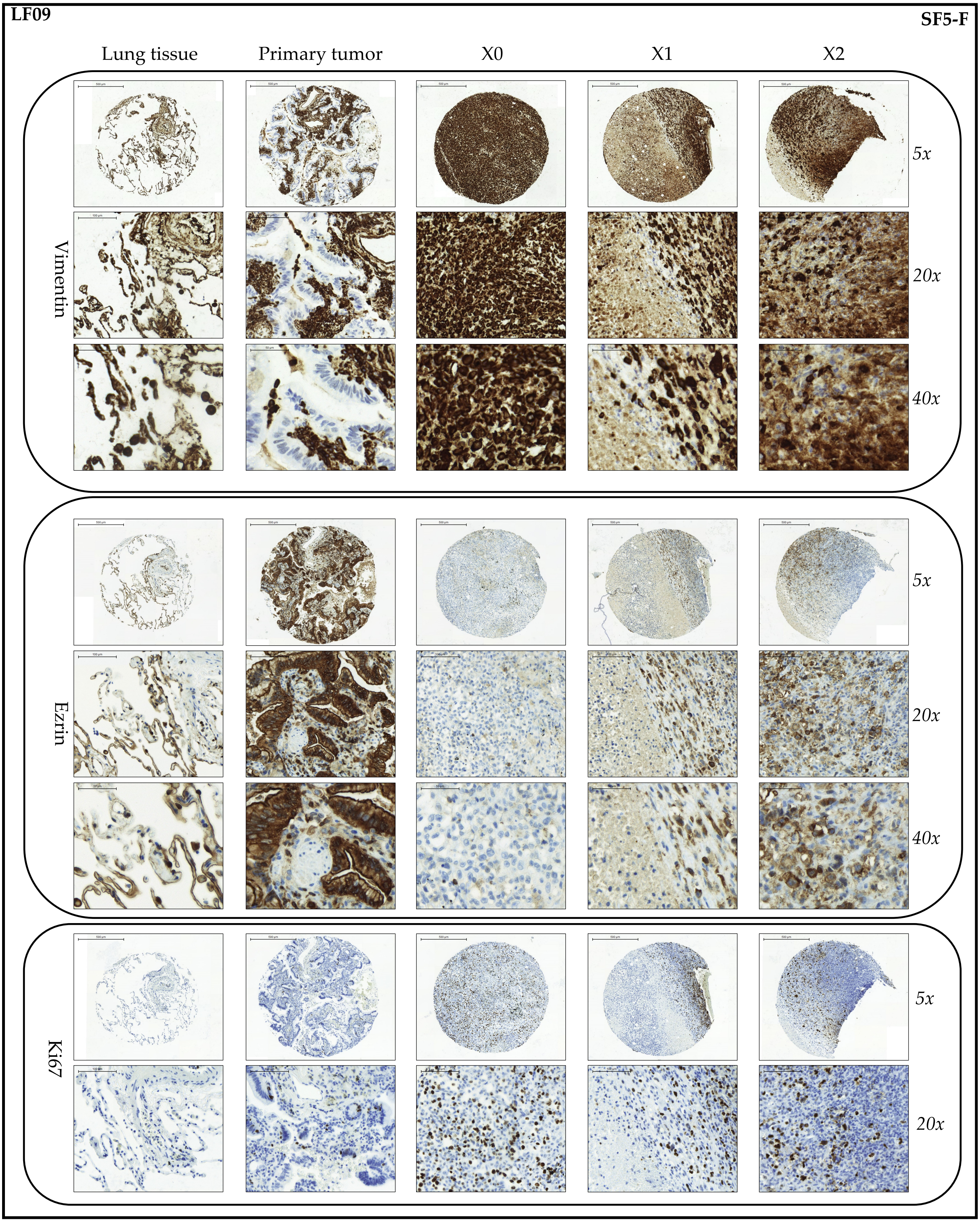

F) LF09: Vimentin, Ezrin and Ki67 panel.

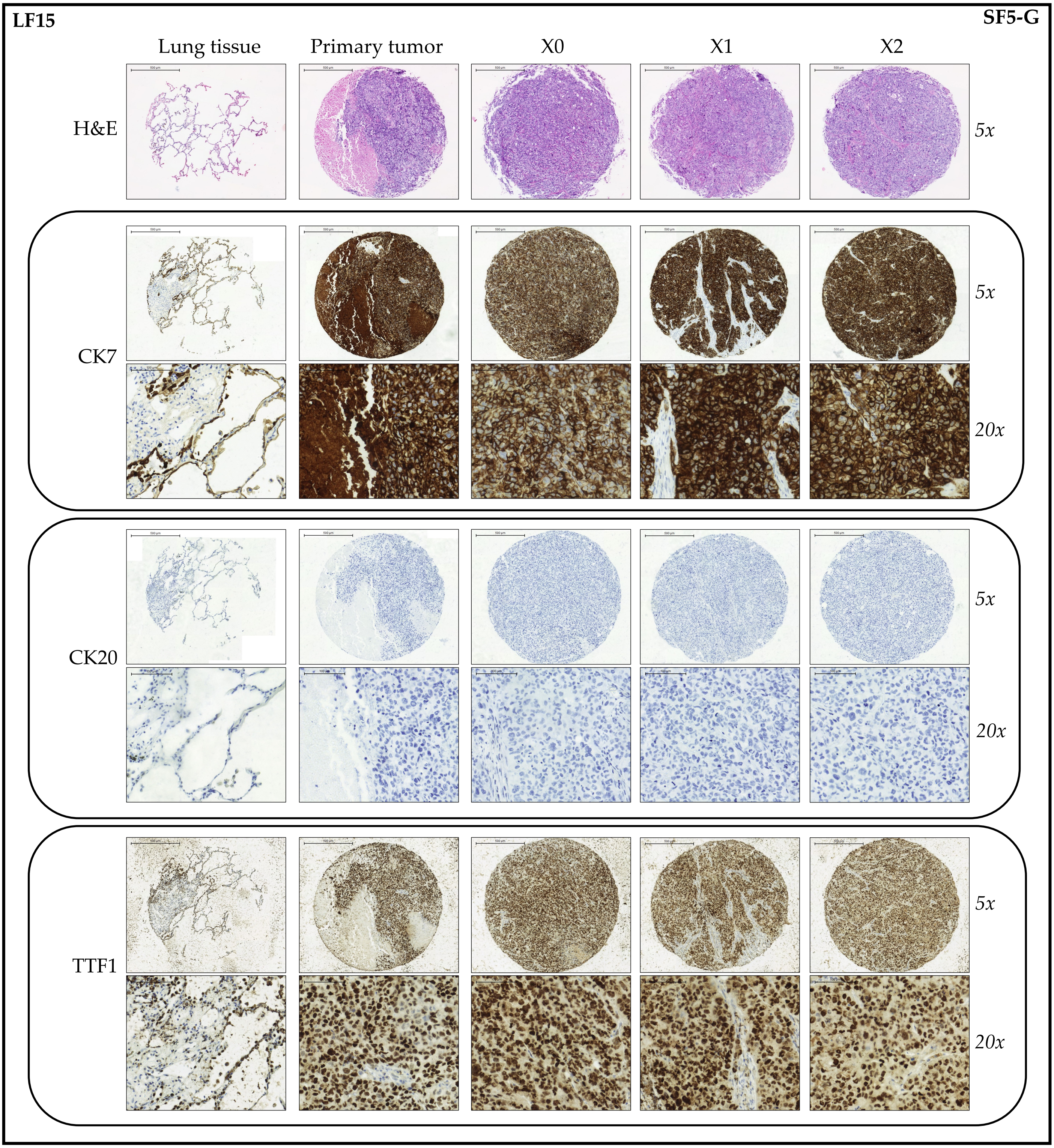

G) LF15: descriptive panel.

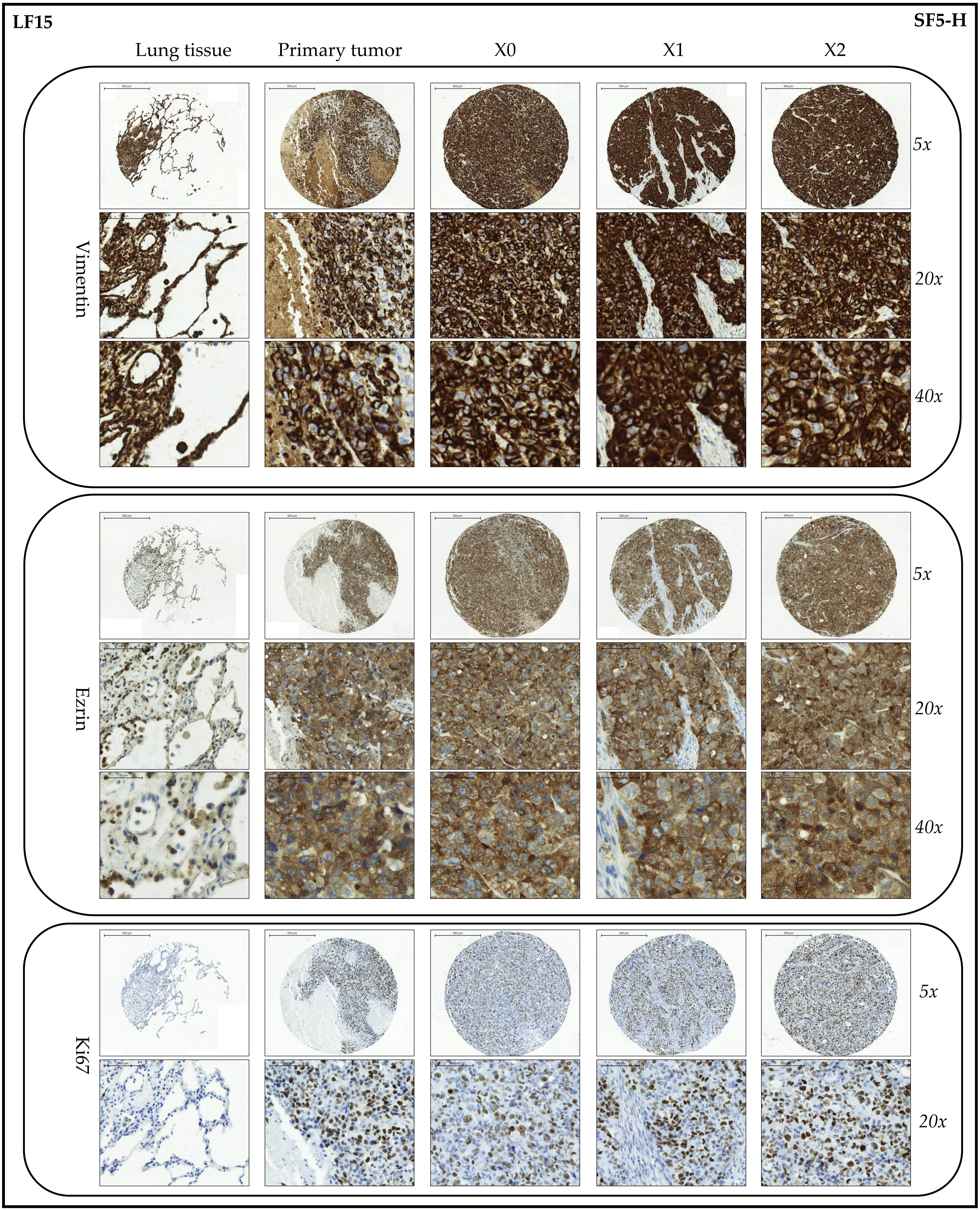

H) LF15: Vimentin, Ezrin and Ki67 panel.

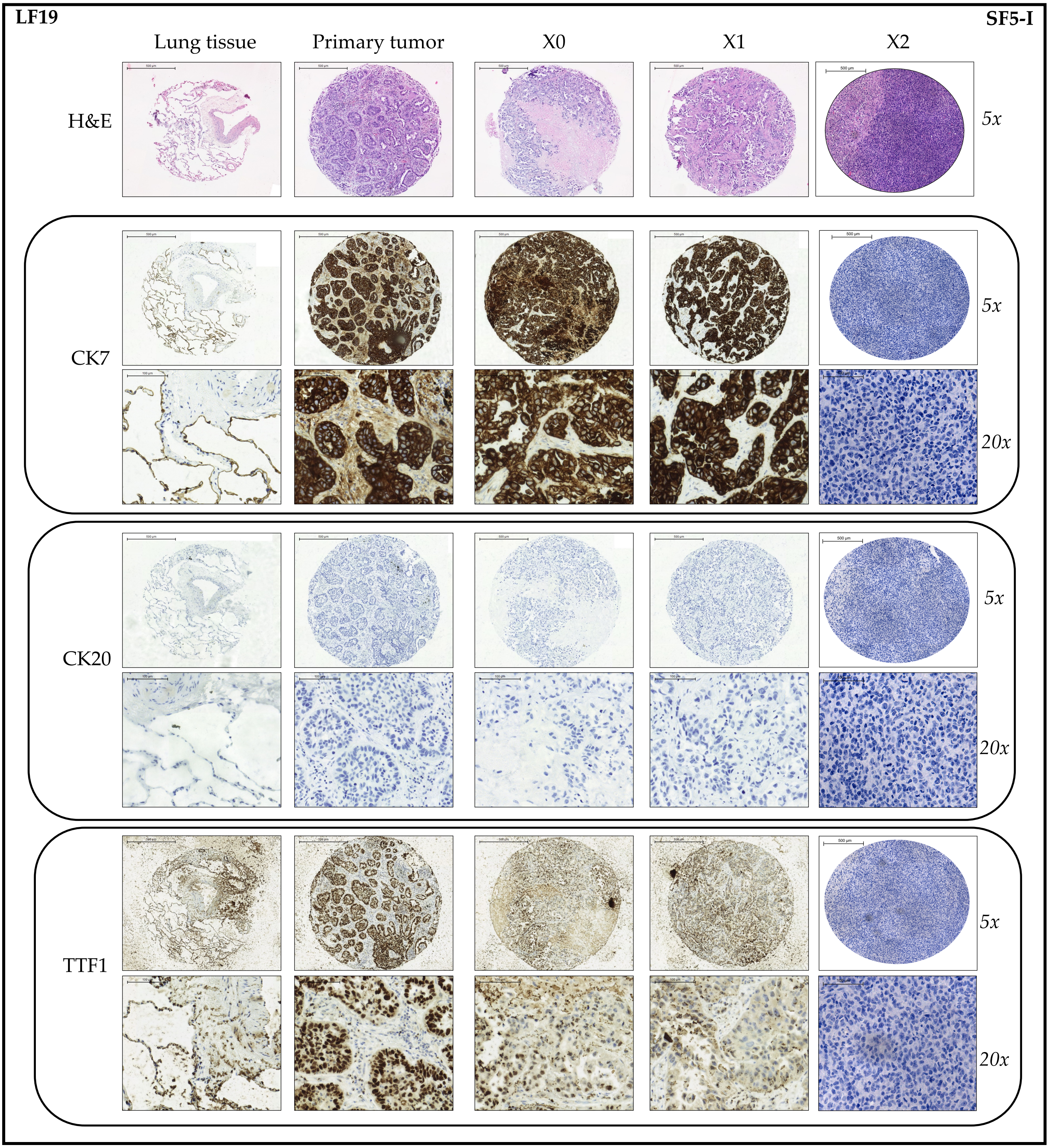

I) LF19: descriptive panel.

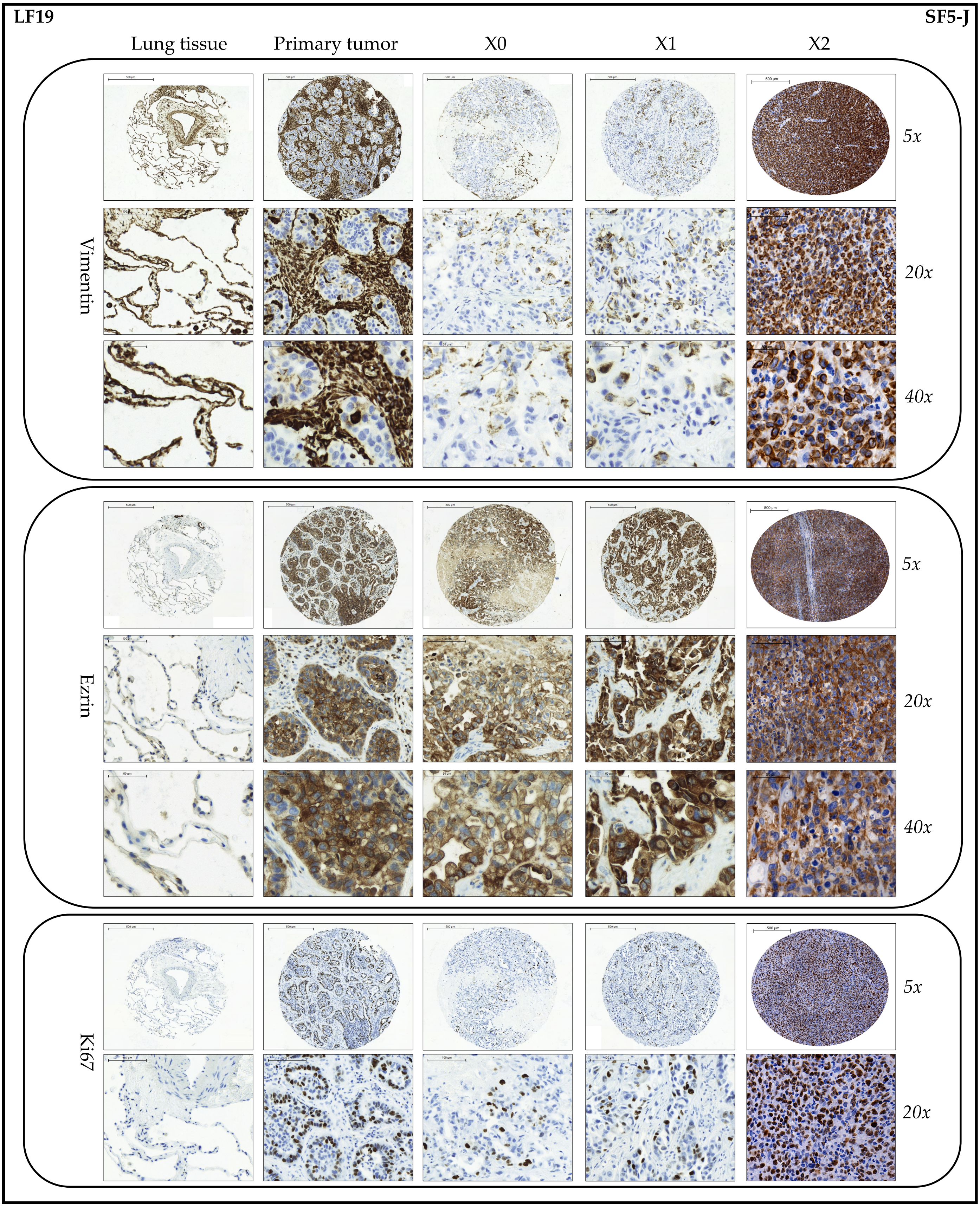

J) LF19: Vimentin, Ezrin and Ki67 panel.

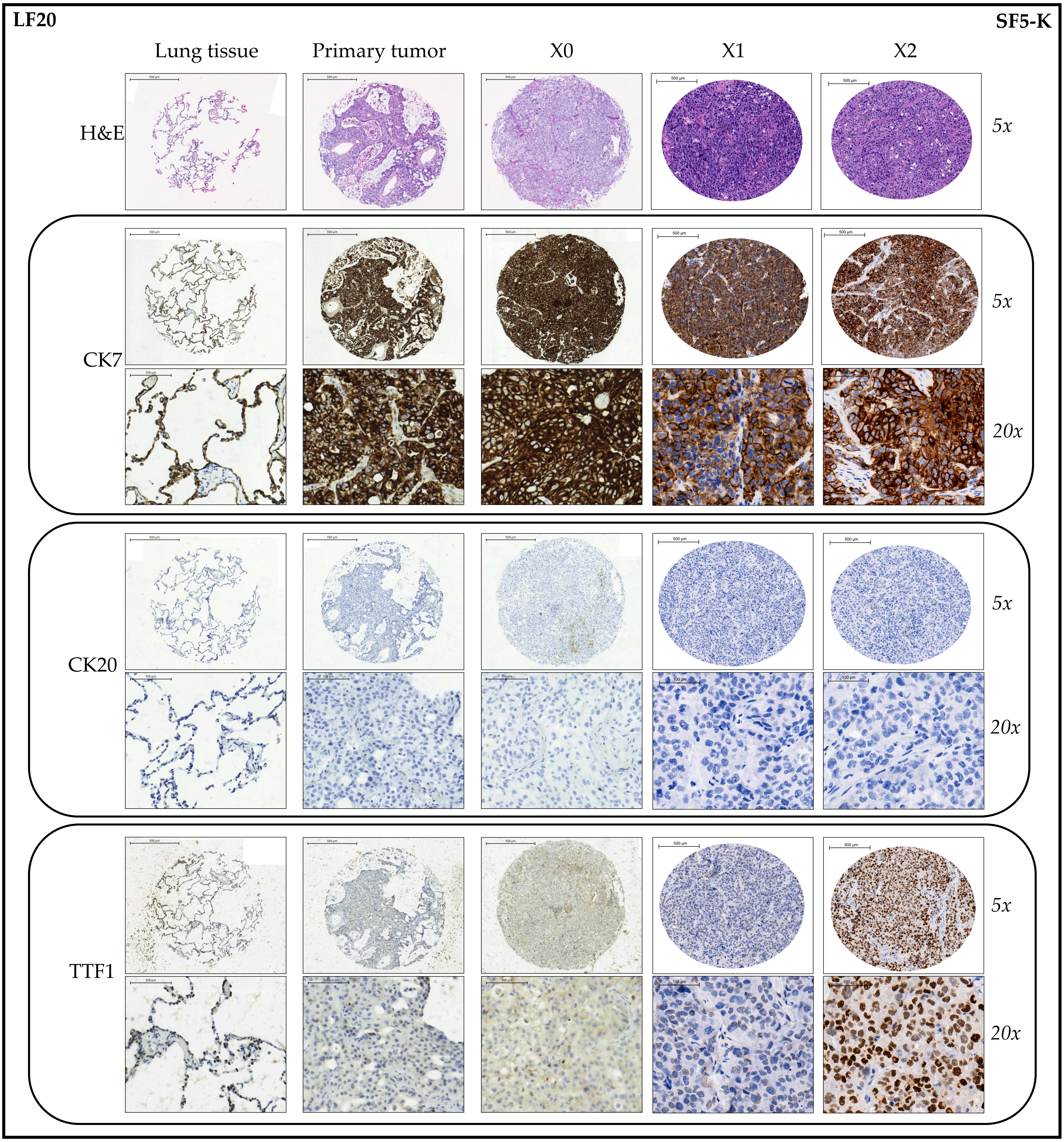

K) LF20: descriptive panel.

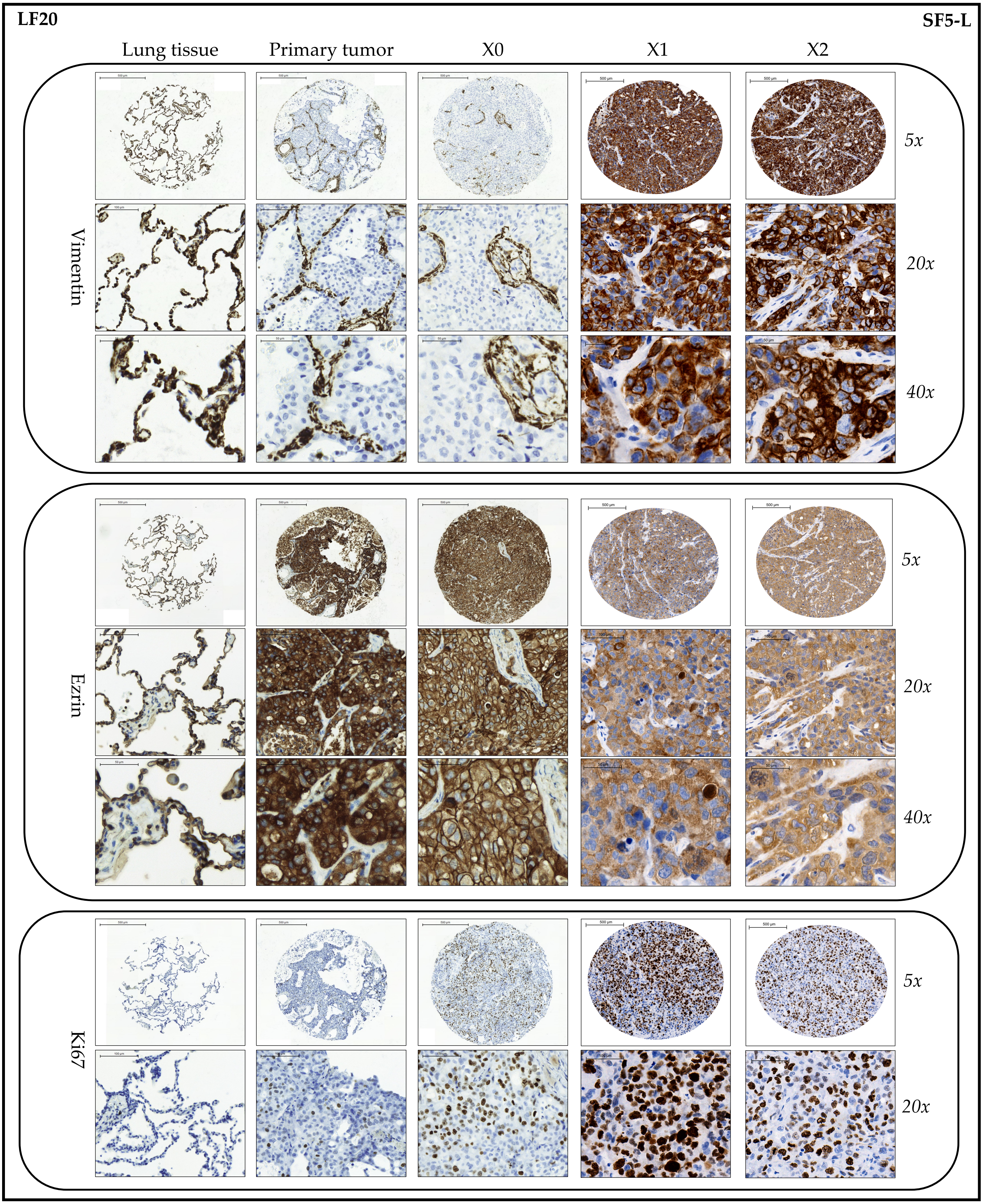

L) LF20: Vimentin, Ezrin and Ki67 panel.

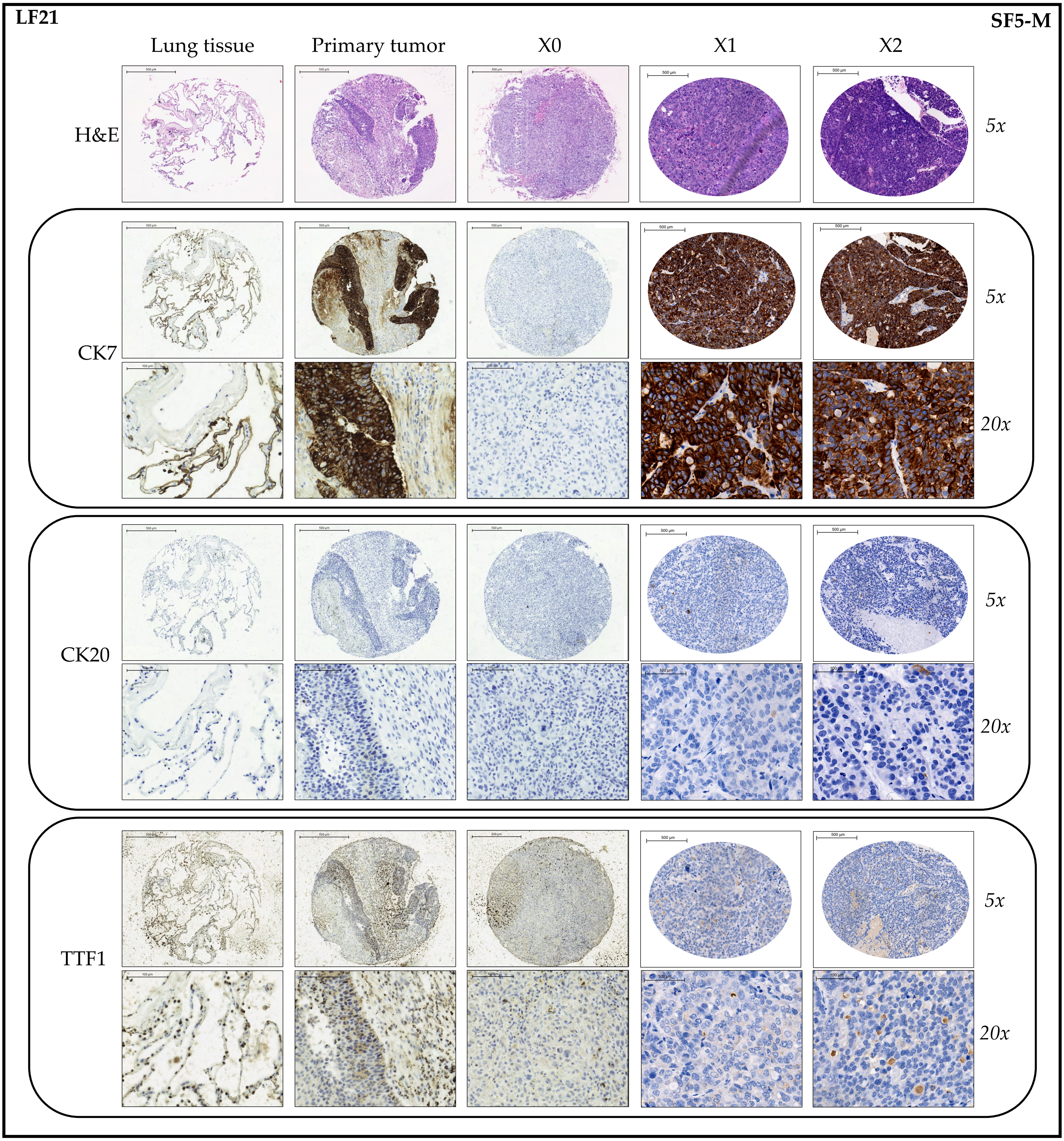

M) LF21: descriptive Ki67 panel.

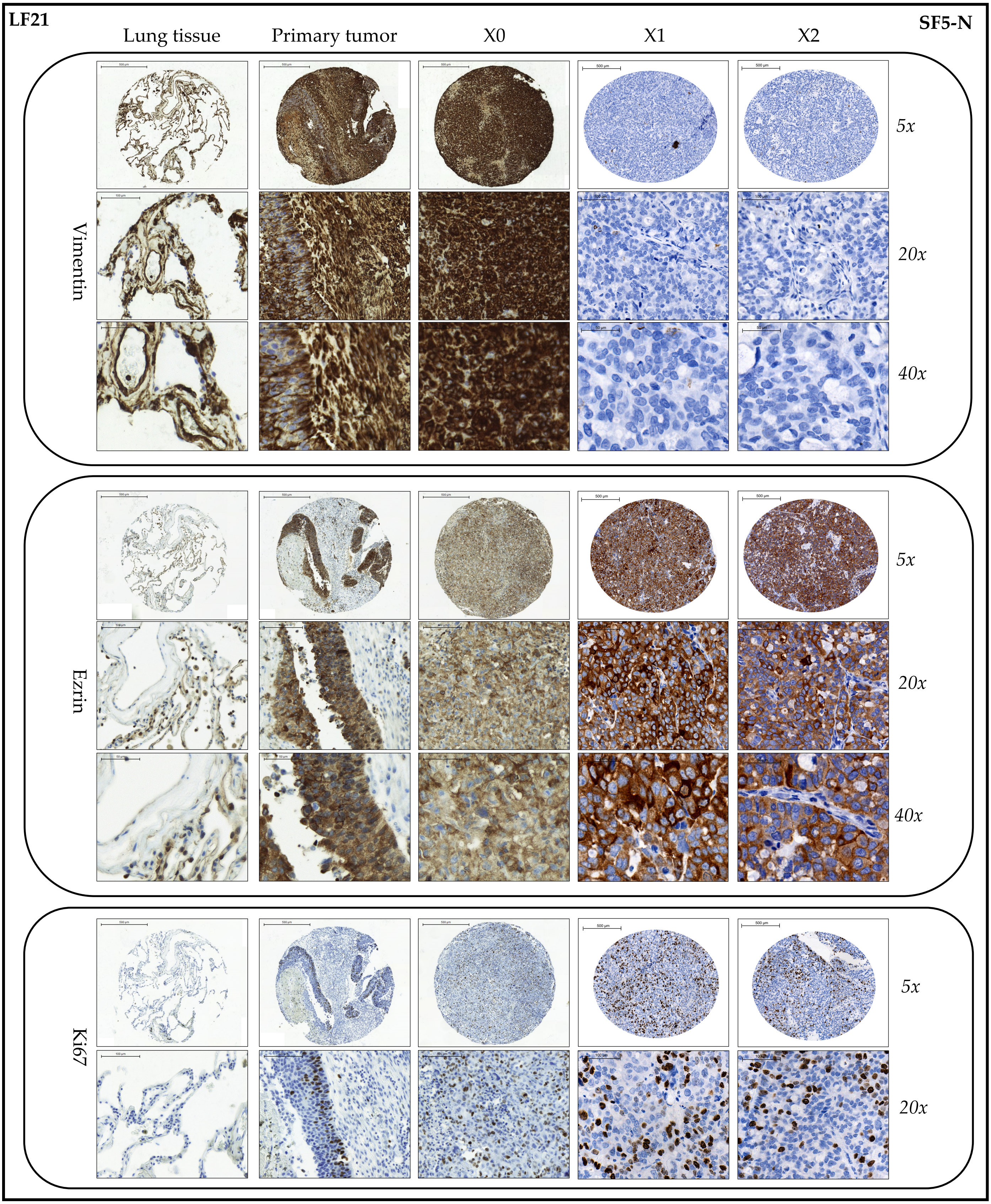

N) LF21: Vimentin, Ezrin and Ki67 panel.

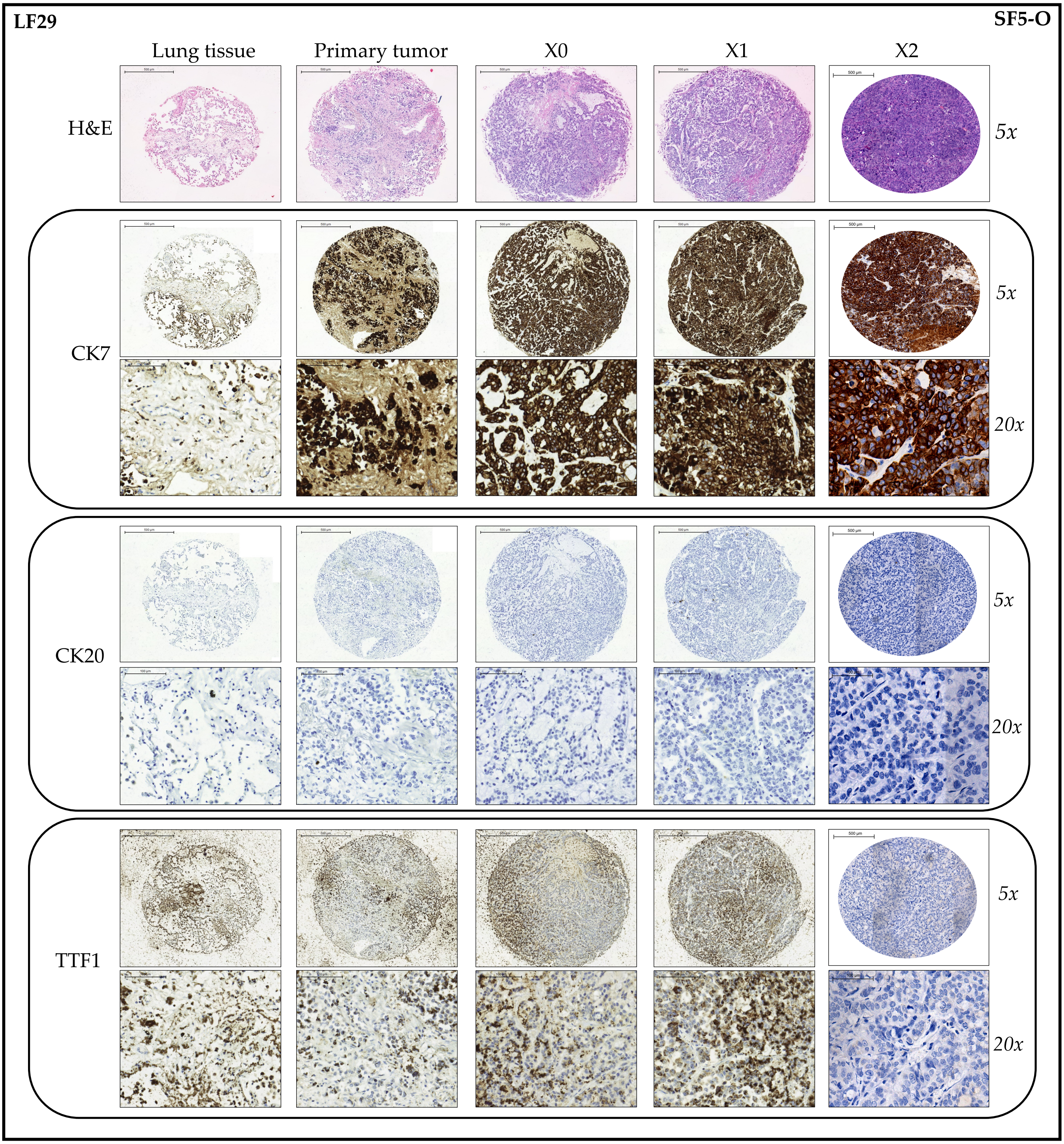

O) LF29: descriptive panel.

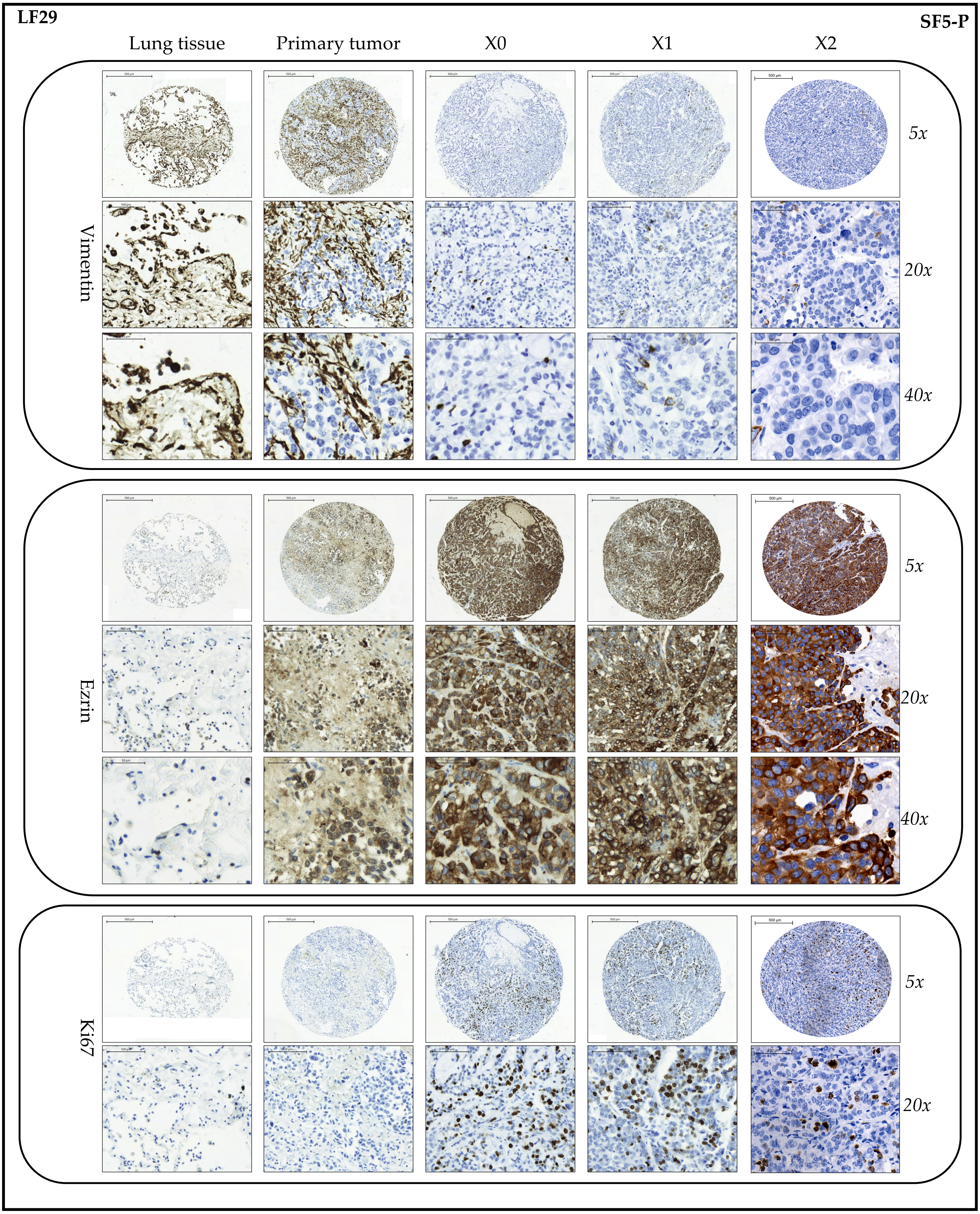

P) LF29: Vimentin, Ezrin and Ki67 panel.

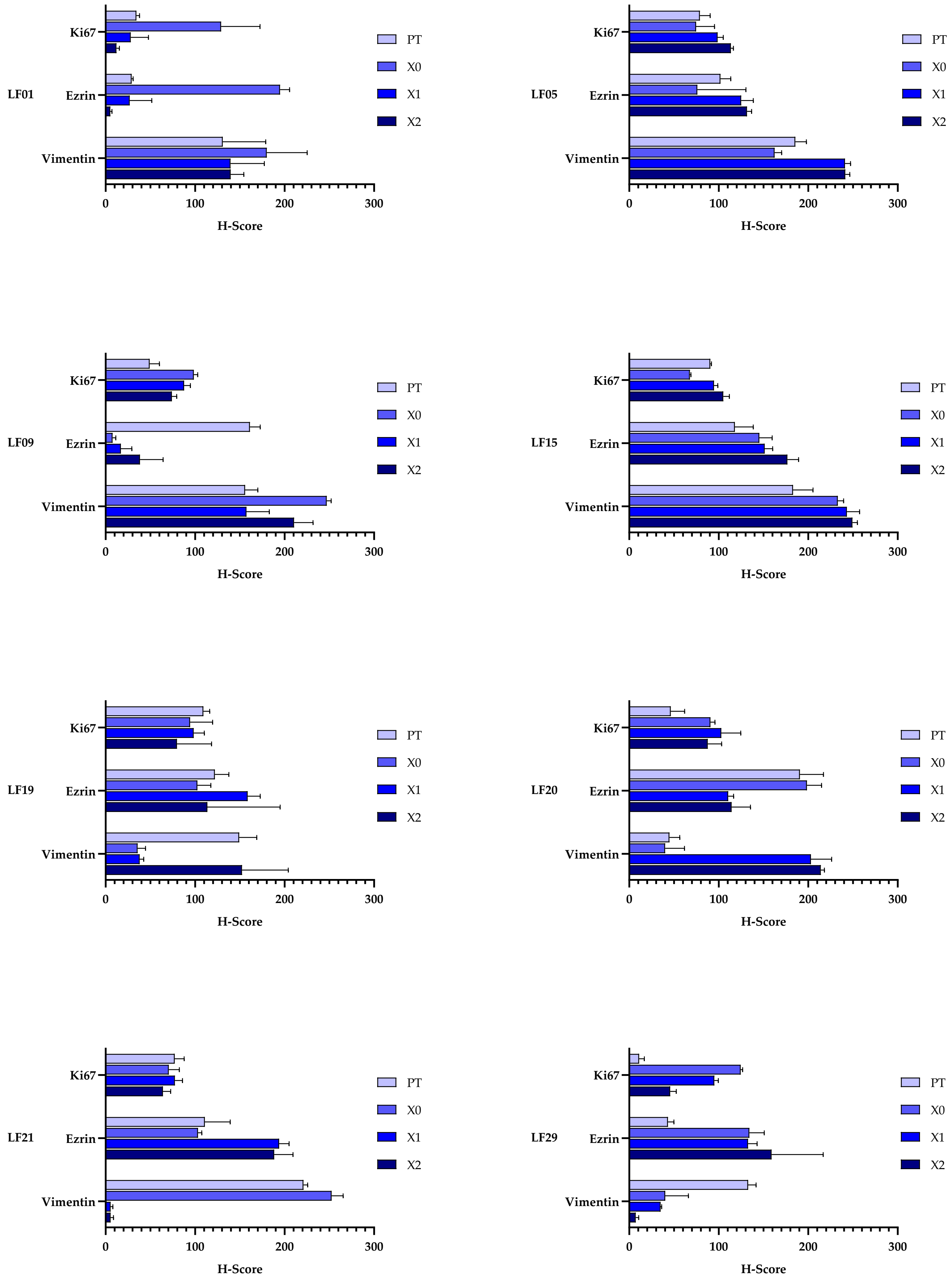

Figure SF6. H-Scores of Vimentin, Ezrin and Ki67 protein expression from the indicated primary tumor (PT) and PDX tumors during passages X0 to X2. Bar charts are shown, with standard deviation.

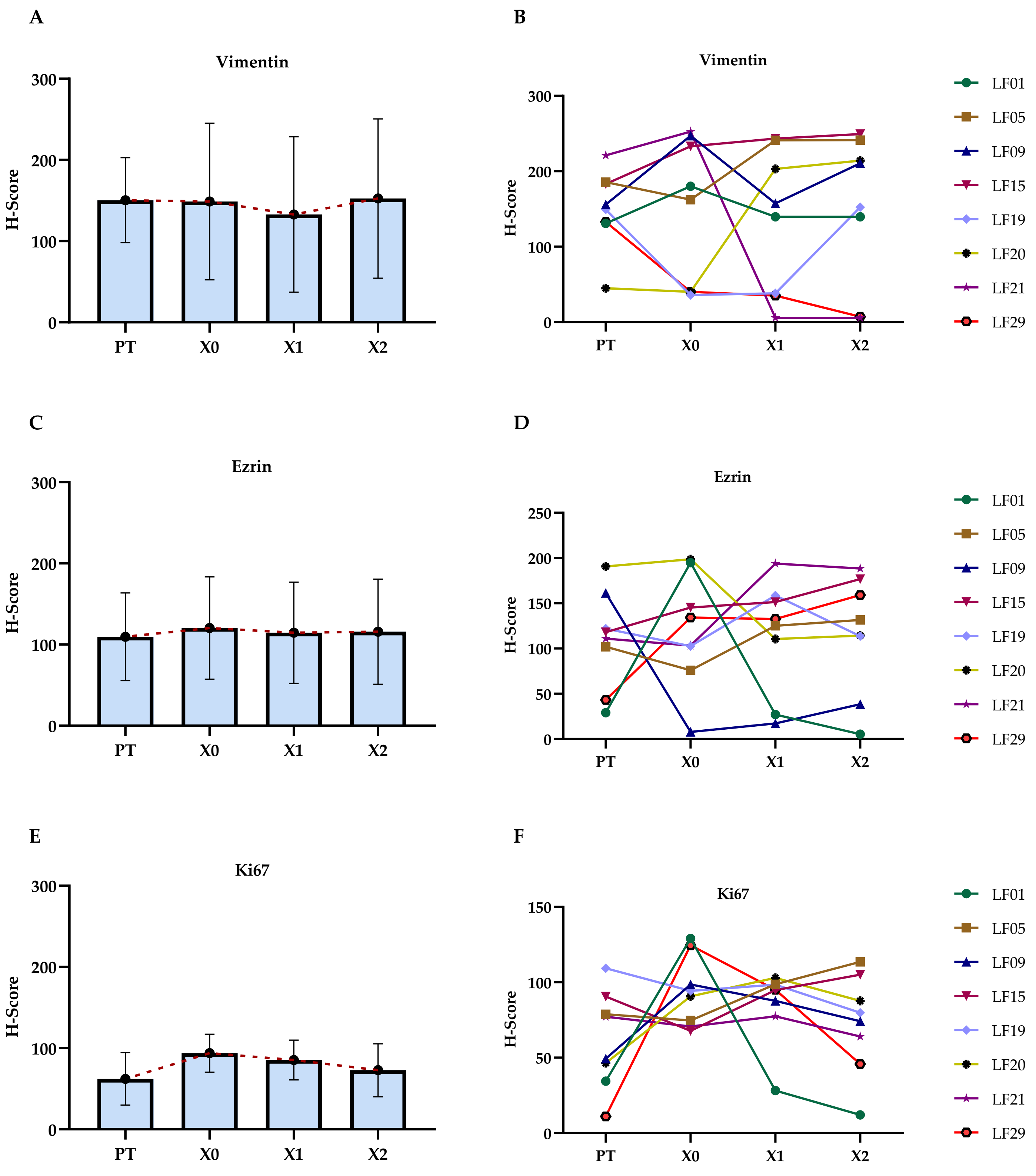

Figure SF7. H-Scores of Vimentin, Ezrin and Ki67 protein expression from all primary tumors (PT) and PDX tumors during passages X0 to X2. (A, C and D) Bar charts of average H-Scores in primary tumor (PT) and passages X0 to X2 samples for the indicated protein are shown. (B,D and F) Protein expression changes analyses by H-Scores in primary tumor (PT) and passages X0 to X2 samples.

		Vimentin		Ezrin		Ki67	
		**H-Score**	**SD**	**H-Score**	**SD**	**H-Score**	**SD**
**LF01**	**PT**	130.98	47.94	30.75	1.78	34.45	3.50
	**X0**	180.10	45.17	180.25	10.74	129.10	43.49
	**X1**	139.53	37.81	32.78	24.72	28.14	19.71
	**X2**	139.72	14.77	6.06	1.78	12.10	3.31
**LF05**	**PT**	185.62	12.35	93.92	11.47	78.87	11.62
	**X0**	162.50	7.71	78.77	54.44	74.68	20.48
	**X1**	241.09	6.16	119.61	13.39	98.70	6.41
	**X2**	241.37	5.12	127.50	5.21	113.50	2.91
**LF09**	**PT**	155.96	14.10	131.23	11.54	49.31	10.81
	**X0**	247.07	5.06	29.42	3.62	98.50	4.43
	**X1**	157.49	25.38	33.87	12.23	87.73	7.06
	**X2**	210.61	21.06	48.55	25.88	74.15	5.21
**LF15**	**PT**	183.11	22.24	111.52	20.40	90.62	1.24
	**X0**	233.07	6.57	125.82	14.31	67.79	1.07
	**X1**	243.25	14.18	138.46	8.87	94.76	4.14
	**X2**	249.33	5.56	157.17	12.51	105.04	6.88
**LF19**	**PT**	149.24	19.62	118.34	15.69	109.26	6.96
	**X0**	35.78	8.78	104.68	15.03	94.35	25.16
	**X1**	38.01	4.64	142.34	14.09	98.46	11.83
	**X2**	152.48	51.64	116.05	81.36	79.81	38.55
**LF20**	**PT**	44.87	11.48	155.96	26.37	46.49	15.35
	**X0**	40.10	21.72	172.95	16.38	90.69	5.12
	**X1**	203.07	23.12	104.35	5.97	102.92	21.71
	**X2**	214.08	3.93	108.98	21.13	87.69	15.56
**LF21**	**PT**	221.08	4.67	100.54	28.41	77.19	10.49
	**X0**	252.59	12.89	94.61	4.21	70.63	11.81
	**X1**	5.6	2.54	167.16	11.19	77.44	8.48
	**X2**	5.52	3.21	158.83	21.05	64.01	8.75
**LF29**	**PT**	132.93	8.85	34.57	6.62	11.04	5.86
	**X0**	40.18	25.88	131.90	16.75	124.62	2.19
	**X1**	35.15	1.17	124.42	10.27	95.05	4.60
	**X2**	7.22	3.33	132.56	57.61	45.74	6.82

Table ST1. H-Scores and standard deviations (SD) of Vimentin, Ezrin and Ki67 protein expression from primary tumors (PT) and PDX tumors during passages X0 to X2.
